# Anticipating the economic resilience of dental practices in crises: a machine learning approach

**DOI:** 10.3389/fpubh.2026.1858605

**Published:** 2026-06-30

**Authors:** Delia Radoi, Dan Curavale, Silviu-Mirel Pituru

**Affiliations:** 1 Department of Organization, Professional Legislation and Dental Office Management, Faculty of Dentistry, Carol Davila University of Medicine and Pharmacy, Bucharest, Romania; 2 National University of Science and Technology POLITEHNICA Bucharest, Bucharest, Romania

**Keywords:** composite indicator, COVID-19, crisis forecasting, dental practices, DPRI, financial resilience, machine learning

## Abstract

**Background/Objectives:**

The COVID-19 pandemic exposed the need for economic resilience in dental practices, which faced reduced patient numbers, increased costs, and service restrictions. This study aimed to develop a predictive model for dental practice resilience and to identify the financial characteristics associated with better performance during and after a crisis.

**Methods:**

We analyzed official financial statements for 2,474 Bucharest dental practices. After applying mode-specific inclusion criteria, the final analytic cohorts comprised 850 firms for crisis-year prediction in 2020 and 801 firms for the three 2021 recovery scenarios. The Dental Practice Resilience Index (DPRI) was developed as a composite measure of profitability, expense efficiency, debt-to-turnover, turnover per employee, and profit per employee. Seven regression model families were evaluated across four prediction scenarios, and continuous predictions were also translated into tertile-based risk classes for decision support.

**Results:**

Crisis-year prediction showed moderate but useful accuracy, with Linear Regression narrowly achieving the highest cross-validated performance (*R*^2^ = 0.537 ± 0.078). Predicting 2021 recovery from pre-crisis data alone was more difficult, and Random Forest narrowly ranked first (*R*^2^ = 0.452 ± 0.084). The strongest scenario-level results were obtained when observed 2020 data were included, with Elastic Net reaching *R*^2^ = 0.643 ± 0.071 and Random Forest reaching balanced accuracy = 0.648 ± 0.044. When observed crisis-year data were unavailable, a two-step synthetic recovery approach recovered part of that information, but remained below the crisis-informed configuration.

**Conclusions:**

Machine learning methods proved effective not only in predicting dental practice resilience during a crisis, but also in anticipating post-crisis recovery potential. The DPRI provided an interpretable resilience measure, while the combination of continuous prediction and tertile-based classification offered both nuanced forecasts and practical risk stratification. Observed crisis-year information substantially improved recovery forecasting, although pre-crisis indicators alone still retained meaningful predictive value.

## 1 Introduction

Major economic crises significantly impact society and inevitably disrupt health systems as well. The COVID-19 pandemic affected service sectors dominated by small and medium-sized enterprises, which experienced abrupt decreases in activity, revenues, and profitability (1, 2). In this context, the ability of a business to remain financially functional under stress became a central concern.

Dentistry was particularly affected by the COVID-19 pandemic because care delivery depends on direct patient contact and regular clinical activity. International evidence has documented severe financial pressure on dental services, while local studies from Bucharest showed reduced patient volume, higher operating costs, staffing adjustments, and uneven financial recovery in 2021 (3–5).

To reduce the impact of future crises, it is important to develop predictive tools that can anticipate which dental practices are more likely to absorb a shock and recover. In this study, economic resilience refers to a practice's ability to withstand a disruption and regain its financial performance after the shock (6). For dental practices, this resilience is reflected not by a single accounting value, but by the joint behavior of profitability, leverage, expense control, and labor productivity. Composite financial indicators provide a practical way to combine such dimensions into interpretable risk and resilience measures (7, 8).

Although the current literature provides descriptive evidence about the financial consequences of COVID-19 for dental services, no previous study has developed a predictive framework tailored to the financial resilience of dental practices. The present study addresses that gap by constructing a composite Dental Practice Resilience Index and forecasting it from historical financial statements.

The framework is positioned at the intersection of four related literatures. First, dental and small-business COVID-19 studies document the sectoral shock and motivate the need for crisis-oriented financial monitoring (1–5). Second, economic and organizational resilience research emphasizes that recovery trajectories are path-dependent and depend on adaptive capacity rather than only on pre-shock resource levels (9–11). Third, composite-indicator and accounting-ratio research provides the methodological basis for translating financial statements into interpretable scoring systems (7, 8). Fourth, machine-learning credit-scoring and default-prediction studies show that flexible algorithms can improve prediction in some financial-risk settings, while also reinforcing the need to compare them against simpler and interpretable benchmarks (12–15). Against this background, the present contribution is not a generic credit-risk model; it adapts financial scoring to dental practices by defining a continuous resilience index and evaluating crisis-year, direct-recovery, crisis-informed, and synthetic-bridge forecasting modes.

The research questions underlying the study were whether pre-crisis financial history can predict resilience during and after a crisis, which engineered financial characteristics drive that resilience most strongly, and how much predictive value is gained when the crisis year is observed directly or approximated from earlier data. We therefore formulated five hypotheses. **H1:** pre-crisis financial history is positively associated with crisis-year DPRI and permits above-baseline prediction of 2020 resilience. **H2:** predicting 2021 recovery from pre-crisis information alone is more difficult than predicting the crisis year because recovery also depends on unobserved shock-year adaptation. **H3:** adding observed 2020 accounting information materially improves 2021 recovery prediction. **H4:** when observed 2020 data are unavailable, a synthetic crisis-year bridge recovers part, but not all, of the predictive value of actual 2020 information. **H5:** interpretable linear or regularized models remain competitive with more flexible ensemble methods because many financial-resilience signals are ratio based and approximately monotonic. To test these hypotheses, four prediction modes were evaluated within a common regression-first framework, with tertile-based risk classes derived from the continuous predictions.

## 2 Materials and methods

### 2.1 Data source and study cohorts

The financial data were extracted from the Romanian Ministry of Finance for firms whose principal activity corresponds to CAEN 8623, dental practice activities. The analytical dataset contains 2,474 firms, and all observations carry Bucharest county code B.

The initial extract was an uncurated administrative file rather than an analysis-ready research cohort. It included firms with no usable financial activity in one or more years, incomplete reporting histories, denominator problems for ratio construction, and invalid accounting entries such as negative debt or non-positive employee counts. The curation procedure was therefore designed to remove observations for which DPRI components or prediction features could not be computed in an internally consistent and verifiable way, rather than to remove firms because of poor financial performance.

For each firm, the yearly variables extracted were turnover, revenue, debt, expenses, profit, and number of employees. The extracted repository file contains harmonized annual columns for 2017–2021; therefore, the empirical endpoint was defined a priori as 2020 for the shock year and 2021 for the first observed recovery year. Later years were not present in the analysis extract and were not used in model fitting. Data processing and model estimation were implemented in Python using pandas and scikit-learn (16, 17).

Table 1 summarizes the accounting fields and curation rules used before feature engineering. These rules were applied before model fitting so that turnover-based ratios, employee-normalized ratios, and debt-based indicators were computed only when their denominators and source values were economically meaningful.

**Table 1:** Accounting variables and preprocessing rules used to construct predictors and DPRI components.

Source field	Operational definition	Cleaning rule
Turnover	Annual turnover used as the denominator for margin, leverage, and efficiency ratios.	Required to be positive in every year needed by a prediction mode.
Profit	Accounting profit or loss used in profit margin and profit per employee.	Negative values were retained because losses are economically meaningful.
Debt	Reported total debt used in the debt-to-turnover ratio.	Required to be present; negative values were treated as invalid and excluded through the complete-case rules.
Expenses	Reported annual expenses used in the expense-efficiency ratio.	Required to be present; ratios were bounded to reduce denominator-driven extremes.
Employees	Average number of employees used in labor-productivity indicators.	Required to be positive before per-employee ratios were computed.

Outlier handling was applied at the ratio level rather than by deleting high- or low-performing firms solely because they were extreme, consistent with composite-indicator practice in which skewed components are bounded or transformed before aggregation (8). Before scaling, profit margin was bounded at [−1, 1], expense efficiency at [0, 2], and debt-to-turnover at [0, 5]. Within each cross-validation split, additional percentile clipping and scaling were fitted only on the training fold and then applied to the validation fold, preventing information leakage. Negative profit was not removed, because losses are part of the resilience construct; negative debt or expense entries were rare and were treated as invalid accounting records.

After applying mode-specific criteria, two final complete-case cohorts remained. The 2020 crisis-year forecasting task used 850 firms with complete 2017–2020 accounting histories. All three 2021 recovery tasks used the same complete-case cohort of 801 firms with complete 2017–2021 histories, so differences between the recovery scenarios reflect information availability rather than sample composition. A cohort-flow audit was added to evaluate selection effects: among 2,474 initially extracted firms, 1,233 had computable 2020 target information and 850 met all complete-case predictor requirements; for 2021, 1,287 had computable target information and 801 met all complete-case predictor requirements. To assess sensitivity to complete-case filtering, a parallel simple-imputation analysis was added using median imputation for numeric predictors within the cross-validation workflow.

The cohort-representativeness audit is therefore part of the dataset-curation record. Complete-case firms were systematically larger and more financially active than excluded firms with incomplete histories. For example, in the crisis-year cohort, included firms had mean 2019 turnover of 809,296 RON and mean 2019 employment of 4.08 employees, compared with 154,510 RON and 0.66 employees among excluded firms with partial 2019 information. Standardized mean differences were largest for employee count (0.55 in 2019 for the crisis-year cohort and 0.53 for the 2021 cohorts), confirming that complete-case analysis should be interpreted as a more stable-reporting practice sample rather than as the full population of registered firms.

### 2.2 Construction of the Dental Practice Resilience Index

The five fundamental metrics described below (Equations 1–5) are informative individually, but they do not fully capture the interdependencies that determine crisis resilience. Therefore, we developed the Dental Practice Resilience Index (DPRI), a composite metric that synthesizes multiple dimensions of financial health into a single interpretable score.


$$\begin{array}{l} \text{Profit Margin}=\frac{\text{Profit}}{\text{Turnover}}\times 100 \end{array}$$ (1)

$$\begin{array}{l} \text{Debt}\text{-to}\text{-Turnover}=\frac{\text{Debt}}{\text{Turnover}} \end{array}$$ (2)

$$\begin{array}{l} \text{Turnover per Employee}=\frac{\text{Turnover}}{\text{Number of Employees}} \end{array}$$ (3)

$$\begin{array}{l} \text{Expense Efficiency}=\frac{\text{Expenses}}{\text{Turnover}} \end{array}$$ (4)

$$\begin{array}{l} \text{Profit per Employee}=\frac{\text{Profit}}{\text{Number of Employees}} \end{array}$$ (5)

Table 2 makes the DPRI construction explicit. Profitability received the largest weight because losses directly reduce liquidity buffers and continuity capacity. Expense efficiency and leverage received the next largest weights because cost rigidity and debt service can amplify revenue shocks. Labor-productivity indicators were retained at lower weights to capture practice scale and workforce efficiency without allowing size alone to dominate the index. The weights were therefore judgment-based but anchored in established accounting-ratio logic and composite-indicator practice (7, 8).

**Table 2:** Dental Practice Resilience Index components, transformations, directions, and weights.

Component	Direction before inversion	Weight	Rationale
Profit margin	Higher is better	0.30	Operating surplus and shock-absorption capacity.
Expense efficiency	Lower is better	0.25	Cost discipline and margin preservation under restrictions.
Debt-to-turnover	Lower is better	0.20	Leverage pressure relative to activity scale.
Turnover per employee	Higher is better	0.15	Labor productivity and revenue generation capacity.
Profit per employee	Higher is better	0.10	Productivity-adjusted profitability; partly overlaps with profit margin.

**Figure 1:**
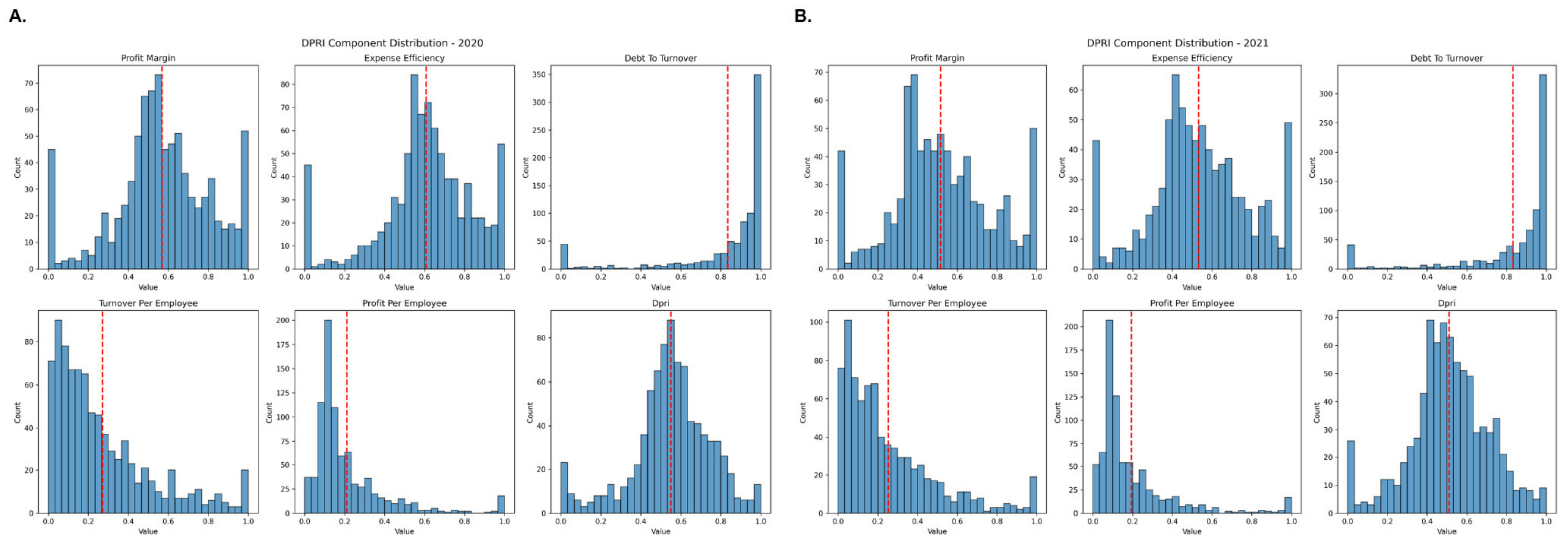
DPRI component distributions after transformation. **(A)** 2020 cohort. **(B)** 2021 cohort.

Prior to transformation, profit margin was clipped to [−1, 1], expense efficiency to [0, 2], and debt-to-turnover to [0, 5]. Turnover per employee and profit per employee were computed only for firms with positive employee counts. Within each evaluation split, additional percentile clipping and scaling were fitted on the training data only. Figure 1 summarizes the transformed DPRI component distributions. As a robustness check on weighting choices, an equal-weight DPRI and leave-one-component-out variants were compared with the primary weighted index. The equal-weight index was almost identical in rank ordering to the primary DPRI (Spearman correlations of 0.994 in 2020 and 0.993 in 2021), indicating that the empirical results are not driven by a fragile weighting scheme.

Combining these established financial indicators into a single index provides a more robust view of a practice's overall financial strength (7, 8). After scaling all five components to a common 0–1 range and inverting the expense-efficiency and debt-to-turnover components so that higher values consistently indicate stronger resilience, DPRI was defined as described in Equation 6:


$$\begin{array}{l} \text{DPRI}=0.30PM_{\text{score}}+0.25EE_{\text{score}}+0.20DT_{\text{score}}\\ \;+\;0.15TPE_{\text{score}}+0.10PPE_{\text{score}}. \end{array}$$ (6)

Higher DPRI values indicate greater financial strength and, therefore, greater resilience to economic shocks.

### 2.3 Feature engineering and feature-policy selection

Beyond the fundamental indicators, the feature space included annual DPRI values and financial ratios from the available historical years, together with engineered variables intended to capture temporal dynamics. These included log-scale terms, revenue and employee growth rates, multi-year stability statistics, binary profitability indicators, interaction terms, and composite indicators such as financial health, operational excellence, and stability score.

**Figure 2:**
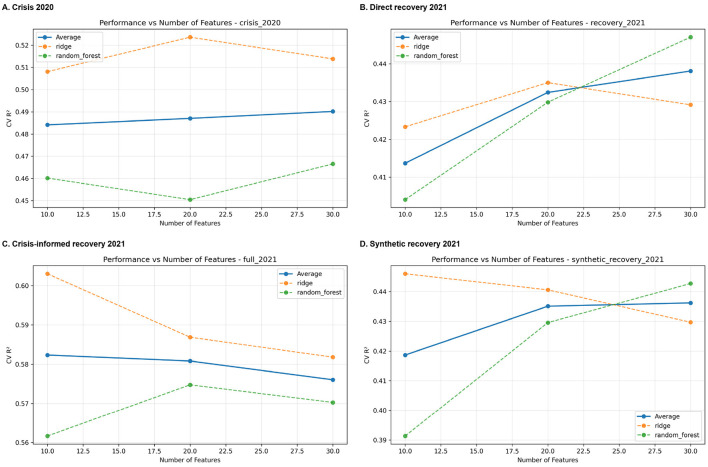
Cross-validated performance vs. number of features across the four prediction modes. **(A)** Crisis-year forecasting. **(B)** Direct recovery forecasting. **(C)** Recovery forecasting with observed 2020 data. **(D)** Synthetic recovery forecasting.

Because the engineered feature space was wide, feature-policy selection was used to compare compact and richer representations fairly. In each outer training split, features were ranked using normalized *F*-statistics, mutual information, and shallow random-forest importance. Candidate subsets of 10, 20, and 30 features were evaluated with Ridge and Random Forest on the training portion only. The reduced branch was retained when its best mean *R*^2^ remained within 0.005 of the all-features branch and its mean balanced accuracy was not worse by more than 0.01; otherwise, the richer branch was kept. Figure 2 summarizes these comparisons.

### 2.4 Prediction modes and evaluation protocol

Four prediction modes were evaluated using distinct labels to avoid ambiguity about predictor and target years. **Crisis-year forecasting** used 2017–2019 predictors to forecast 2020 DPRI. **Direct recovery forecasting** used the same pre-crisis 2017–2019 predictor window to forecast 2021 DPRI, deliberately excluding 2020. **Crisis-informed recovery forecasting** used 2017–2020 predictors to forecast 2021 DPRI. **Synthetic crisis-bridge recovery forecasting** first estimated 2020 DPRI from 2017–2019 information and then appended this fold-wise predicted 2020 signal to the pre-crisis feature set for the 2021 forecast.

All reported models were trained as regressors on continuous DPRI and compared in a common configuration set: Linear Regression, Ridge, Lasso, Elastic Net, Random Forest, Extra Trees, and Gradient Boosting. Linear models used StandardScaler, whereas tree-based ensembles used RobustScaler. The study therefore compares model families within a common evaluation protocol rather than through exhaustive nested hyperparameter tuning.

**Figure 3:**
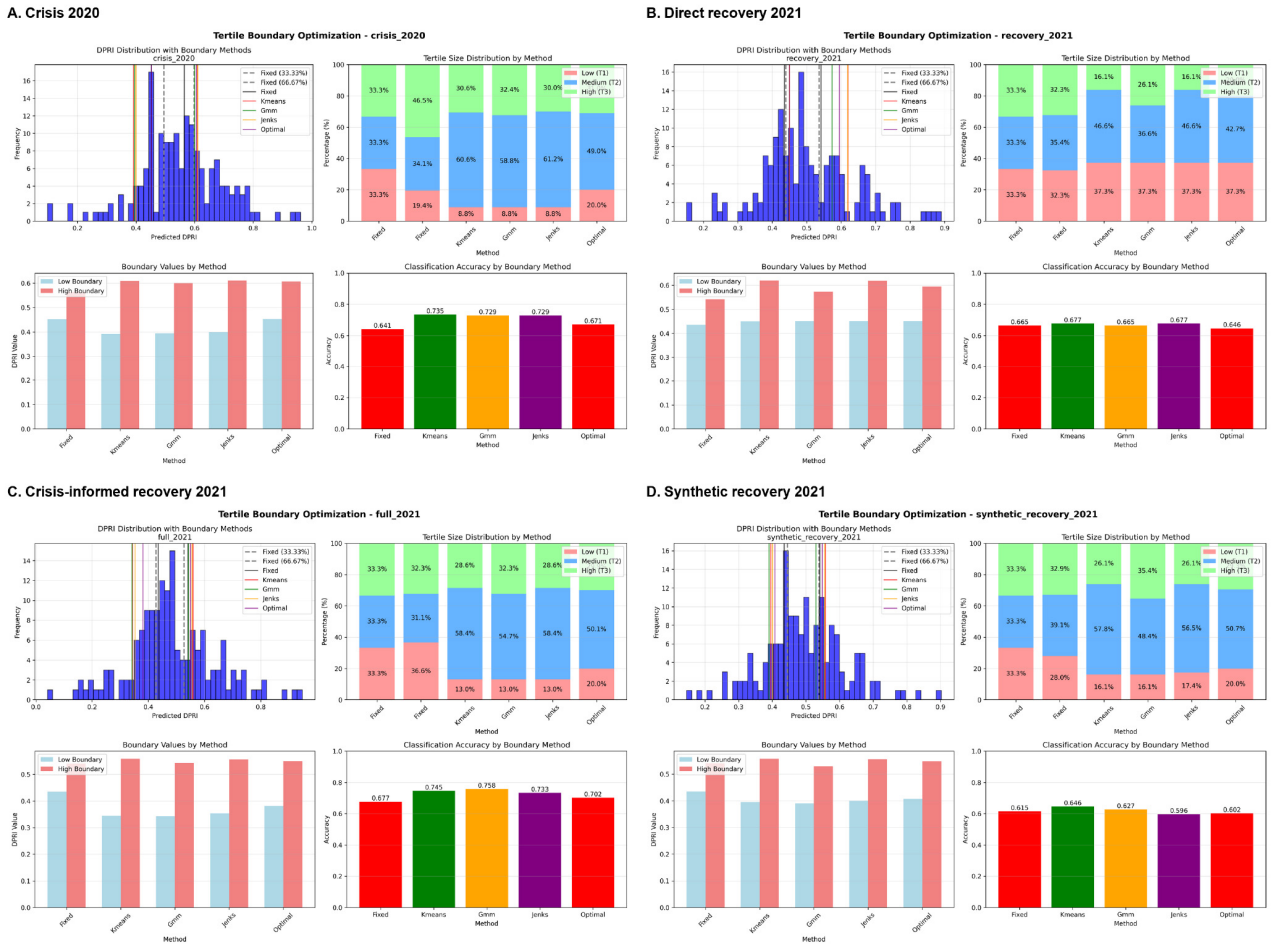
Training-time tertile-boundary behavior across the four prediction modes. **(A)** Crisis-year forecasting. **(B)** Direct recovery forecasting. **(C)** Recovery forecasting with observed 2020 data. **(D)** Synthetic recovery forecasting.

Classification was derived post hoc by converting actual and predicted DPRI values into three ordered tertiles (T1–T3). Within each outer training fold, a three-component Gaussian mixture model was fitted to the training DPRI values and the intersections between adjacent components were used as boundaries. When the Gaussian-mixture fit was unstable, Jenks natural breaks served as fallback. Figure 3 presents the corresponding boundary behavior.

The primary tables report mean and standard deviation over five-fold outer cross-validation. Figures were generated from a fixed 80/20 test split and are included to illustrate model behavior visually. Regression performance is reported with *R*^2^, RMSE, and MAE, while derived classification performance is reported with Accuracy, Balanced Accuracy, weighted *F*1, and Cohen's Kappa. Because several models produced similar scores, additional sensitivity and interpretability checks were conducted: paired fold-level model comparisons, repeated-seed and subsample robustness, complete-case vs. simple-imputation sensitivity, TreeSHAP attribution for Random Forest benchmark models (18), model-agnostic permutation importance, finite-difference driver effects, and accounting-ratio baseline comparisons. These checks were used to support cautious interpretation rather than to replace the primary cross-validation tables.

## 3 Results

The results are presented for the four prediction modes described above. Cross-validation tables provide the main basis for model comparison, whereas the fixed 80/20 split is shown only to illustrate model behavior visually. Accordingly, a figure may present the model that performed best on that split even when a different model ranks first in cross-validation. Additional diagnostics, feature-policy curves, boundary values, and extended test-split tables are reported in the Supplementary material.

### 3.1 Regression performance

#### 3.1.1 Crisis-period prediction

Table 3 presents the regression performance metrics for crisis-year forecasting. Linear Regression narrowly ranked first by cross-validated *R*^2^ (0.537 ± 0.078), while Ridge was essentially tied at the same rounded mean. These results show that pre-crisis accounting structure contains useful, although moderate, signal for forecasting 2020 resilience; they should not be read as evidence that one linear specification is statistically superior to the others.

**Table 3:** Regression performance metrics for crisis-year forecasting (2020).

Model	*R*^2^ mean	*R*^2^ SD	RMSE mean	RMSE SD	MAE mean	MAE SD
Linear Regression	0.5369	0.078	0.1328	0.0133	0.0939	0.0095
Ridge	0.5369	0.0778	0.1328	0.0132	0.0941	0.0093
Lasso	0.5363	0.064	0.1333	0.0142	0.0958	0.0102
Elastic Net	0.5354	0.0731	0.1332	0.014	0.095	0.01
Random Forest	0.4994	0.0806	0.1382	0.0139	0.0985	0.0093
Gradient Boosting	0.4817	0.1015	0.1401	0.0139	0.0996	0.008
Extra Trees	0.4607	0.0943	0.1431	0.0135	0.1024	0.0095

#### 3.1.2 Recovery prediction without 2020 data

Table 4 summarizes the regression results for direct recovery prediction without observed 2020 data. Random Forest narrowly ranked first with *R*^2^ = 0.452 ± 0.084, followed closely by Elastic Net and Lasso. The narrow separation between these models suggests that forecasting 2021 recovery from pre-crisis information alone is intrinsically more difficult and that the leading models are best interpreted as practically comparable.

**Table 4:** Regression performance metrics for direct recovery forecasting (2021) without 2020 data.

Model	*R*^2^ mean	*R*^2^ SD	RMSE mean	RMSE SD	MAE mean	MAE SD
Random Forest	0.4522	0.084	0.1452	0.0158	0.1056	0.012
Elastic Net	0.4483	0.0836	0.1458	0.0158	0.107	0.0116
Lasso	0.4461	0.0841	0.146	0.0159	0.108	0.0113
Extra Trees	0.4435	0.0908	0.1464	0.017	0.1056	0.0124
Ridge	0.4262	0.102	0.1486	0.0185	0.1077	0.0134
Linear Regression	0.4207	0.1052	0.1493	0.0189	0.108	0.0139
Gradient Boosting	0.4095	0.0995	0.1506	0.0165	0.1091	0.0123

#### 3.1.3 Recovery prediction with observed crisis data

Table 5 presents the strongest regression results of the study. When observed 2020 information was included, Elastic Net narrowly ranked first with *R*^2^ = 0.643 ± 0.071, followed closely by Lasso and Random Forest. The improvement over direct recovery prediction confirms that crisis-year performance contains substantial information about post-crisis recovery, while the small gap among the leading models argues for cautious model-ranking language.

**Table 5:** Regression performance metrics for recovery forecasting (2021) with observed 2020 data.

Model	*R*^2^ mean	*R*^2^ SD	RMSE mean	RMSE SD	MAE mean	MAE SD
Elastic Net	0.6434	0.0713	0.1169	0.0146	0.0852	0.0085
Lasso	0.6428	0.0661	0.1171	0.0138	0.0862	0.0081
Random Forest	0.6252	0.0798	0.1199	0.0163	0.0853	0.0108
Gradient Boosting	0.6136	0.088	0.1216	0.0168	0.0865	0.0107
Extra Trees	0.6101	0.0868	0.1222	0.0171	0.0866	0.0098
Ridge	0.5975	0.1056	0.1238	0.0195	0.0883	0.0122
Linear Regression	0.5886	0.1119	0.1251	0.0201	0.0897	0.0124

#### 3.1.4 Recovery prediction using synthetic 2020 data

Table 6 reports the results for the synthetic recovery approach, in which predicted 2020 DPRI is used in place of observed crisis-year information. Elastic Net narrowly ranked first with *R*^2^ = 0.451 ± 0.086, with Lasso and Random Forest close behind. A predicted crisis-year signal therefore carries meaningful information, but it does not fully substitute for observed 2020 outcomes.

**Table 6:** Regression performance metrics for synthetic recovery forecasting (2021).

Model	*R*^2^ mean	*R*^2^ SD	RMSE mean	RMSE SD	MAE mean	MAE SD
Elastic Net	0.4508	0.0865	0.1454	0.0162	0.1054	0.0118
Lasso	0.4492	0.0854	0.1456	0.016	0.1068	0.0114
Random Forest	0.4469	0.0911	0.146	0.0176	0.1056	0.0122
Extra Trees	0.432	0.0766	0.148	0.0156	0.1065	0.0101
Gradient Boosting	0.4088	0.1038	0.1508	0.0182	0.1083	0.0119
Ridge	0.4038	0.1247	0.1514	0.0211	0.1084	0.0158
Linear Regression	0.3902	0.128	0.1531	0.0215	0.1095	0.0161

**Figure 4:**
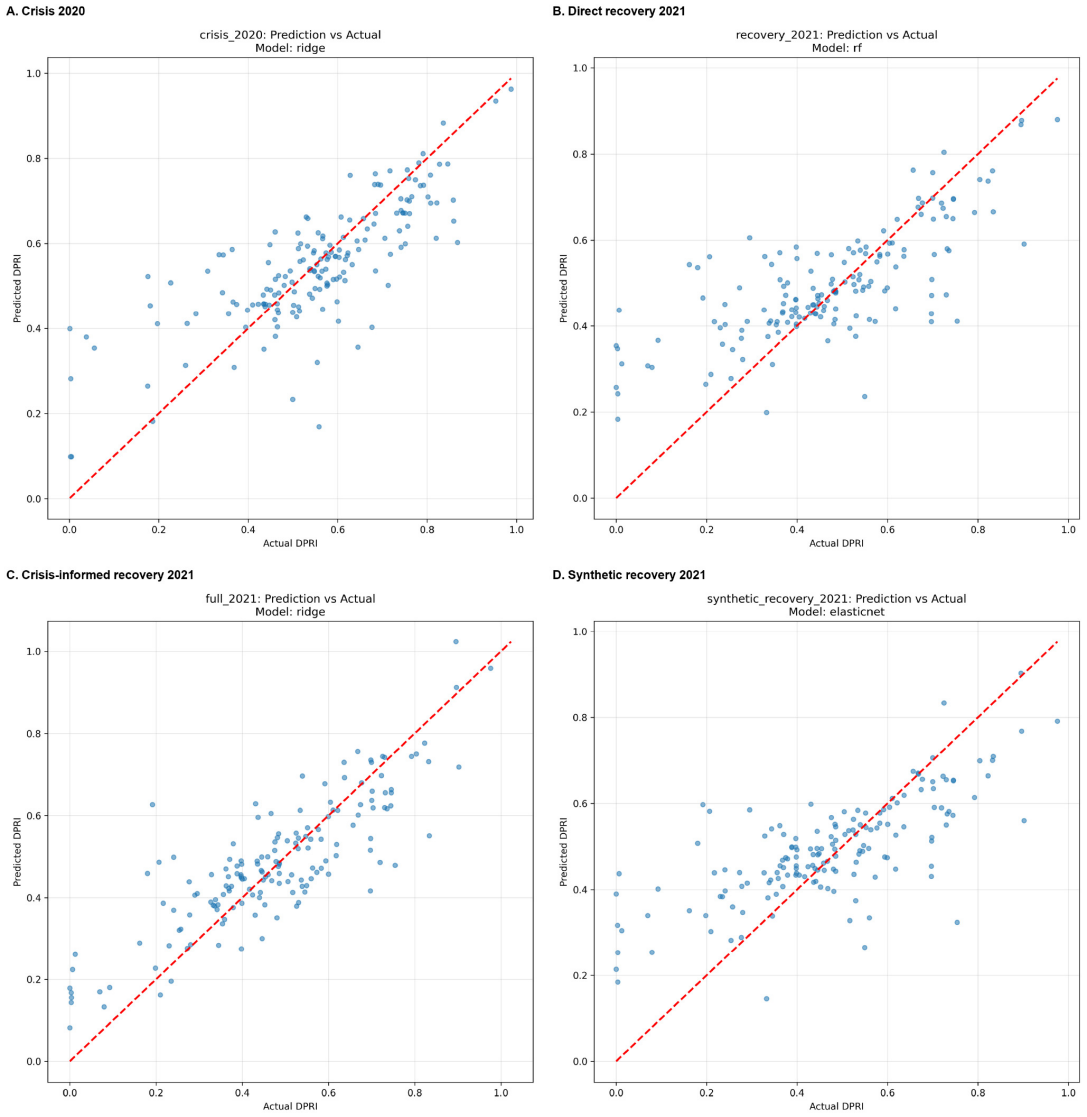
Prediction-vs.-actual DPRI plots on the illustrative fixed 80/20 test split. **(A)** Crisis-year forecasting. **(B)** Direct recovery forecasting. **(C)** Recovery forecasting with observed 2020 data. **(D)** Synthetic recovery forecasting.

**Figure 5:**
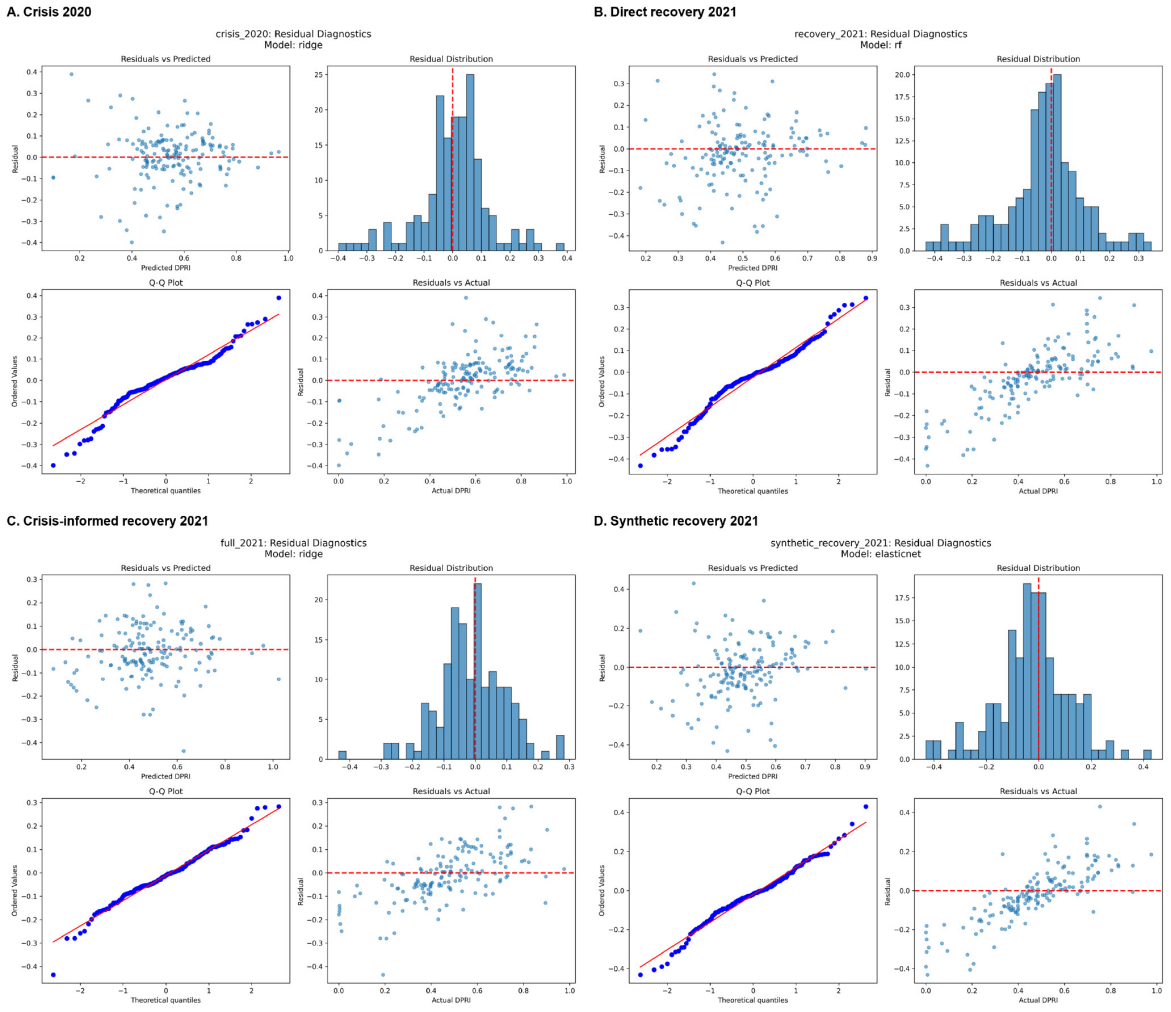
Residual diagnostics on the illustrative fixed 80/20 test split. **(A)** Crisis-year forecasting. **(B)** Direct recovery forecasting. **(C)** Recovery forecasting with observed 2020 data. **(D)** Synthetic recovery forecasting.

On the illustrative fixed 80/20 splits, prediction-vs.-actual plots showed the tightest point cloud for the crisis-informed recovery mode, followed by crisis-year prediction and the two recovery modes that do not use observed 2020 outcomes (Figure 4). Residual diagnostics remained centered near zero across all four modes, with the lowest residual dispersion in the crisis-informed recovery configuration (Figure 5).

### 3.2 Classification performance

#### 3.2.1 Crisis-period classification

Table 7 reports classification performance derived from crisis-year DPRI regression predictions. Linear Regression narrowly achieved the strongest crisis-mode balanced accuracy (0.628 ± 0.022). The narrow spread among the leading models suggests that modest differences in continuous fit do not translate into large differences once predictions are converted to tertiles.

**Table 7:** Classification performance derived from regression predictions for crisis-year forecasting (2020).

Model	Accuracy mean	Accuracy SD	Balanced acc. mean	Balanced acc. SD	*F*1 mean	*F*1 SD	Kappa mean	Kappa SD
Linear Regression	0.6471	0.0153	0.6277	0.0218	0.6473	0.016	0.4512	0.0286
Ridge	0.6424	0.0221	0.6219	0.0292	0.6424	0.0222	0.4435	0.0397
Elastic Net	0.6459	0.0363	0.6206	0.0453	0.6449	0.0375	0.4471	0.0598
Lasso	0.6447	0.038	0.6199	0.0465	0.6441	0.0384	0.4458	0.0619
Random Forest	0.6294	0.0298	0.6158	0.0343	0.6332	0.0304	0.4279	0.0536
Gradient Boosting	0.6294	0.0271	0.615	0.0374	0.6318	0.0276	0.4275	0.0434
Extra Trees	0.6224	0.0205	0.6119	0.03	0.6255	0.0213	0.4179	0.0372

#### 3.2.2 Recovery-period classification without 2020 data

Table 8 reports classification performance for direct recovery forecasting without observed 2020 data. Linear Regression ranked first by cross-validated balanced accuracy (0.587 ± 0.033), but the difference from Ridge and Extra Trees was small. The overall level of performance remained below the crisis-informed configuration, which is consistent with the absence of crisis-year information.

**Table 8:** Classification performance derived from regression predictions for direct recovery forecasting (2021) without 2020 data.

Model	Accuracy mean	Accuracy SD	Balanced acc. mean	Balanced acc. SD	*F*1 mean	*F*1 SD	Kappa mean	Kappa SD
Linear Regression	0.593	0.0365	0.5873	0.0328	0.602	0.0359	0.3887	0.0524
Ridge	0.5917	0.0399	0.5872	0.0353	0.6009	0.0386	0.388	0.0561
Extra Trees	0.5904	0.057	0.5848	0.058	0.6009	0.0571	0.3834	0.0868
Random Forest	0.5805	0.0426	0.5795	0.0413	0.5921	0.0405	0.3723	0.062
Lasso	0.5805	0.0339	0.5788	0.0293	0.5892	0.0325	0.3738	0.046
Gradient Boosting	0.5718	0.0519	0.5712	0.0437	0.5802	0.0482	0.3606	0.0698
Elastic Net	0.5742	0.0358	0.569	0.0341	0.5836	0.0338	0.3632	0.0494

#### 3.2.3 Recovery classification with observed crisis data

Table 9 shows the strongest classification results of the study. Random Forest ranked first in cross-validation with balanced accuracy = 0.648 ± 0.044, closely followed by Lasso and Elastic Net. The result mirrors the regression findings: once the actual crisis-year state is observed, both continuous prediction and tertile grouping become materially easier.

**Table 9:** Classification performance derived from regression predictions for recovery forecasting (2021) with observed 2020 data.

Model	Accuracy mean	Accuracy SD	Balanced acc. mean	Balanced acc. SD	*F*1 mean	*F*1 SD	Kappa mean	Kappa SD
Random Forest	0.6667	0.0384	0.6476	0.0437	0.6742	0.0347	0.4917	0.0588
Lasso	0.6592	0.0392	0.6457	0.0421	0.6675	0.0345	0.4836	0.0586
Elastic Net	0.6592	0.0354	0.6454	0.0307	0.6677	0.0293	0.4838	0.0491
Gradient Boosting	0.6617	0.0425	0.6388	0.048	0.6686	0.0386	0.4835	0.0663
Extra Trees	0.6529	0.0353	0.6314	0.0446	0.6593	0.0341	0.4698	0.0568
Ridge	0.6479	0.0261	0.6304	0.0313	0.6561	0.0242	0.4647	0.0405
Linear Regression	0.6367	0.0282	0.6175	0.0314	0.6458	0.0271	0.4477	0.0436

#### 3.2.4 Recovery classification using synthetic crisis data

Table 10 reports the synthetic recovery classification results. Random Forest ranked first in cross-validation with balanced accuracy = 0.589 ± 0.033, again with limited practical separation from the other leading models. This level was close to direct recovery prediction but remained below the configuration that used observed 2020 data.

**Table 10:** Classification performance derived from regression predictions for synthetic recovery forecasting (2021).

Model	Accuracy mean	Accuracy SD	Balanced acc. mean	Balanced acc. SD	*F*1 mean	*F*1 SD	Kappa mean	Kappa SD
Random Forest	0.5905	0.0391	0.5889	0.0325	0.6007	0.0366	0.3867	0.0523
Extra Trees	0.5855	0.0424	0.5829	0.0444	0.5938	0.0413	0.3767	0.0635
Elastic Net	0.588	0.0396	0.5816	0.0397	0.596	0.0377	0.3812	0.0571
Gradient Boosting	0.5793	0.0269	0.5798	0.0177	0.5884	0.0255	0.37	0.0347
Lasso	0.5855	0.0369	0.5796	0.0369	0.5946	0.0352	0.3781	0.0534
Ridge	0.578	0.0408	0.5736	0.0378	0.5874	0.0384	0.3663	0.0589
Linear Regression	0.573	0.0352	0.5682	0.0312	0.5839	0.0321	0.3599	0.0491

**Figure 6:**
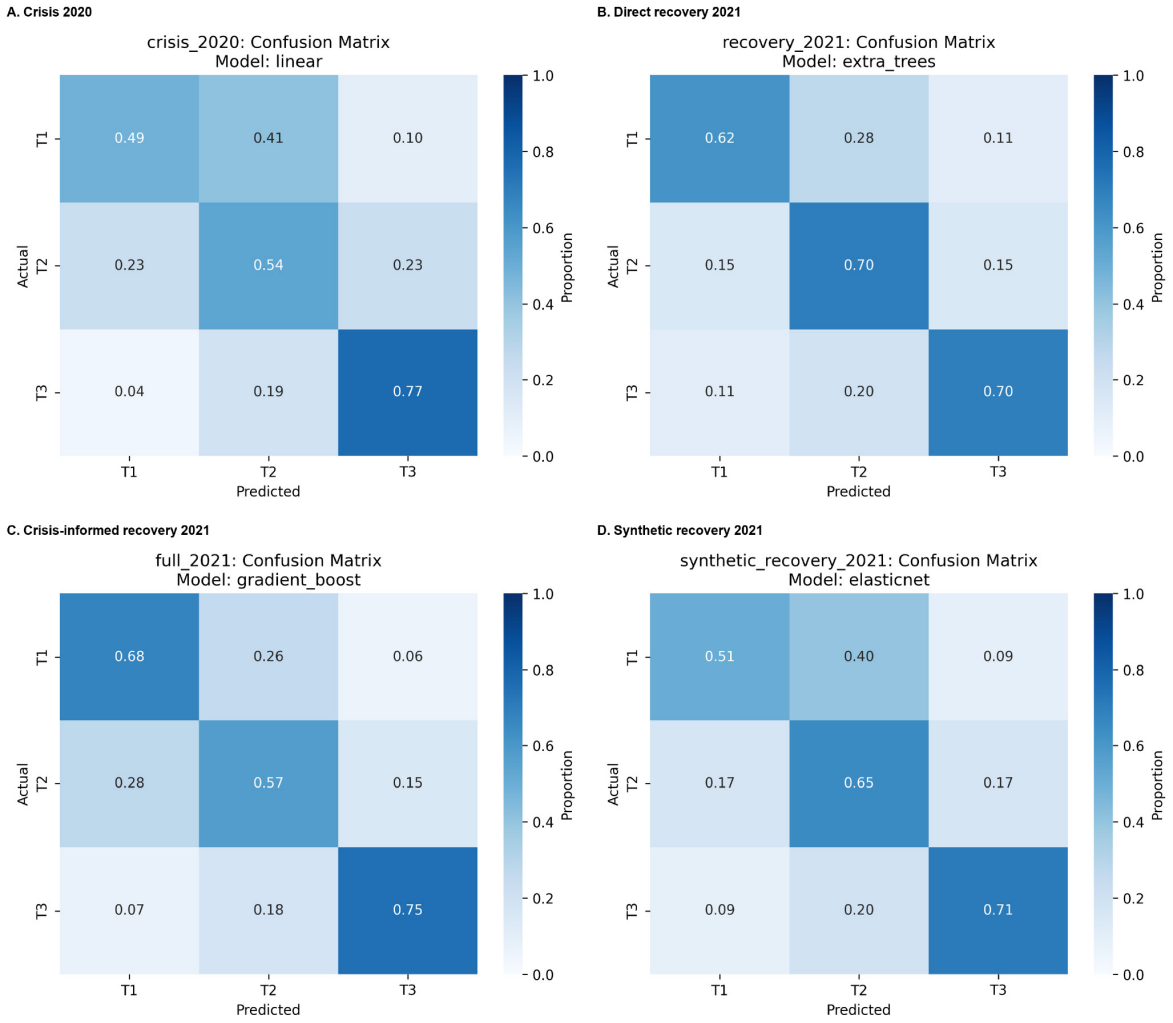
Confusion matrices for tertile classifications derived from regression predictions on the illustrative fixed 80/20 test split. **(A)** Crisis-year forecasting. **(B)** Direct recovery forecasting. **(C)** Recovery forecasting with observed 2020 data. **(D)** Synthetic recovery forecasting.

The confusion matrices in Figure 6 show that the models generally identify the extreme tertiles more reliably than the middle tertile. This pattern is consistent with the continuous nature of DPRI and the information loss introduced by tertile discretization.

### 3.3 Synthesis of the four prediction modes

Taken together, the four scenarios reveal three clear patterns. First, the crisis-informed recovery mode is the strongest overall configuration, reaching *R*^2^ = 0.643 ± 0.071 and balanced accuracy = 0.648 ± 0.044. Second, the synthetic recovery approach recovers part of the missing crisis-year signal, but it does not match the configuration that uses observed 2020 accounting values. Third, reduced feature representations were sufficient for the crisis and direct recovery tasks, whereas richer feature sets were more useful for the broader 2021 representations.

**Figure 7:**
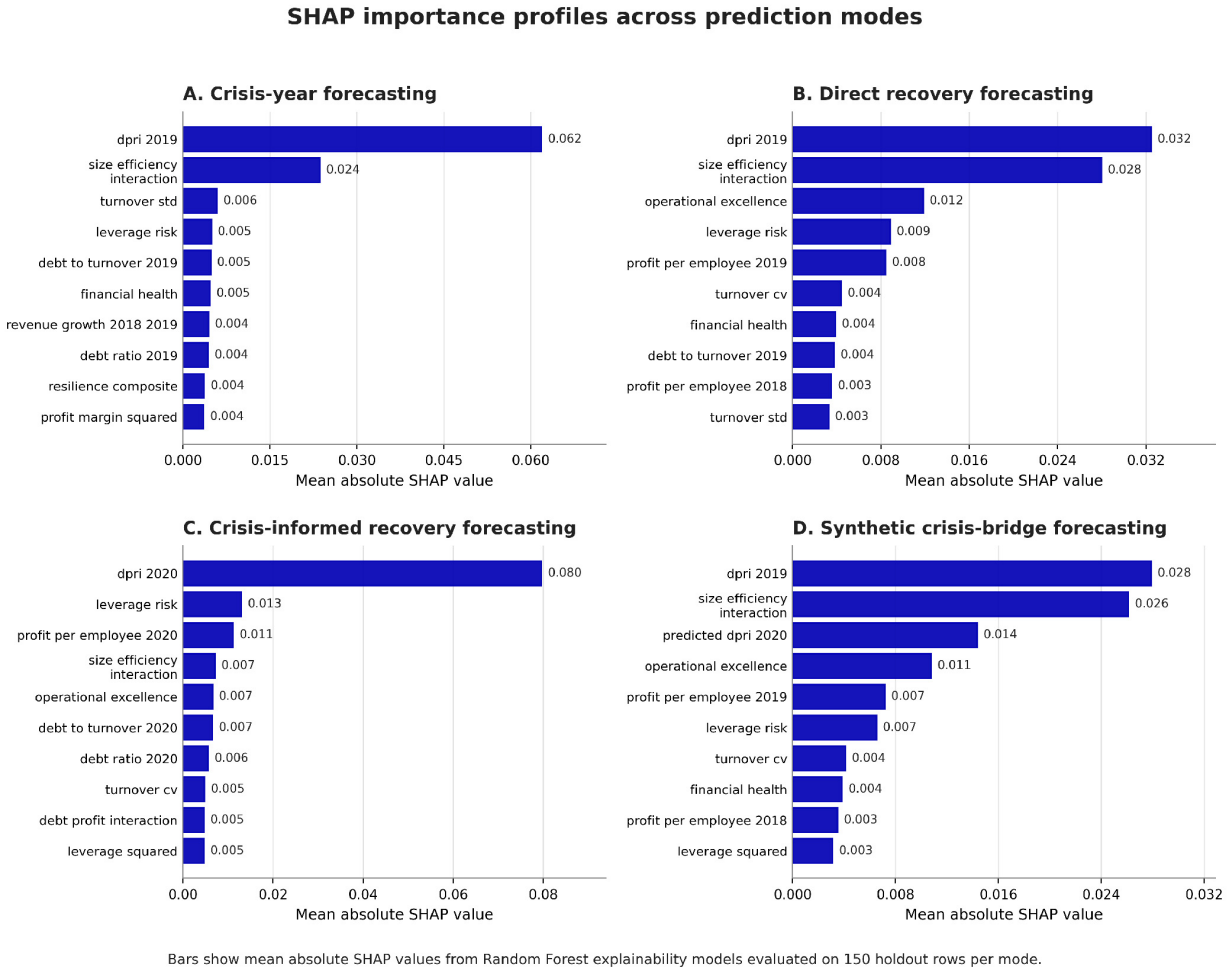
SHAP feature-attribution profiles for the four prediction modes. Bars report mean absolute SHAP values from Random Forest explainability models evaluated on 150 holdout rows per mode. **(A)** Crisis-year forecasting. **(B)** Direct recovery forecasting. **(C)** Recovery forecasting with observed 2020 data. **(D)** Synthetic recovery forecasting.

The SHAP attribution profiles support the same interpretation while providing a model-explainability view of the financial drivers (Figure 7). DPRI in 2019 had the largest mean absolute SHAP value in the crisis-year and direct-recovery modes, indicating that the most recent pre-crisis resilience score carried substantial predictive information. When observed 2020 information was included, DPRI in 2020 became the dominant attribution signal, reinforcing the path-dependent interpretation that crisis-period performance conditions immediate recovery. In the synthetic recovery mode, predicted 2020 DPRI was among the leading SHAP-ranked features, but pre-crisis DPRI and the size-efficiency interaction remained slightly stronger, showing that the synthetic bridge recovered part of the crisis-year signal without replacing the underlying pre-crisis financial structure.

### 3.4 Model comparison, explainability, and robustness checks

The complete methodological audit tables and figures are reported in Supplementary Appendix A.8 to keep the main manuscript focused on the core predictive findings. The dataset-curation checks, including cohort flow, included-vs.-excluded comparisons, cleaning counts, and simple-imputation sensitivity, are linked to the cohort definitions in the Materials and methods. The remaining model-focused checks showed narrow differences among the leading algorithms, supporting cautious language rather than claims of absolute superiority. The main SHAP figure, together with supplementary permutation-importance and finite-difference analyses, supported the same interpretation: recent DPRI, leverage, efficiency, and productivity signals carry the principal predictive information. Accounting-ratio baseline comparisons showed that standard financial ratios remain competitive benchmarks, while repeated-seed and subsample robustness checks confirmed that the central conclusions were stable across resampling choices.

## 4 Discussion

These findings have important implications for dental practice management and financial planning, especially in preparing for future economic disruptions. The DPRI provides a multidimensional measure of financial resilience and, at the same time, offers a structured way to discuss how profitability, leverage, operating efficiency, and stability contribute to that resilience.

The strongest empirical finding is the value of observed crisis-year information. Recovery forecasting improved materially when 2020 variables were included, and DPRI in 2020 became the dominant feature in the crisis-informed 2021 mode. This is consistent with the broader small-business resilience literature, which suggests that post-crisis recovery depends not only on pre-shock capacity, but also on how firms perform and adapt during the shock itself (19–21).

This pattern can be interpreted through path dependency and adaptive capacity (9–11). Pre-crisis profitability, leverage, and productivity define the starting position from which a practice enters the shock, but 2020 performance records how that starting position was translated into actual adaptation under restrictions, patient-volume changes, and cost pressure. The large gain from including observed 2020 data therefore suggests that recovery is not only a function of static pre-crisis strength; it is conditioned by crisis-period behavior, which becomes the immediate platform for 2021 recovery.

The comparison between model families is also informative. Crisis-year forecasting was led by linear models, direct recovery by Random Forest in regression but by Linear Regression after discretization, and crisis-informed recovery by Elastic Net or Random Forest depending on the metric. No single algorithm dominated every task, and the added paired comparisons showed very small *R*^2^ gaps among leading models. This supports a cautious interpretation aligned with the contemporary credit-scoring, bankruptcy-prediction, and SME-default-prediction literature: flexible machine-learning models can improve prediction in some settings, but simpler and regularized benchmarks remain important when the signal is largely financial, ratio-based, and monotonic (12–15).

### 4.1 Practical and policy implications

From a practical point of view, these results support periodic DPRI-based monitoring as part of routine financial planning. Because the most important predictors consistently involved profitability, leverage, efficiency, and stability, the index can help practice owners organize attention around the financial dimensions that matter most for resilience. The continuous DPRI scale is useful for tracking deterioration or improvement over time, whereas tertile classes provide a simpler communication layer for screening and prioritization.

A practical implementation would function as an early-warning system rather than as an automatic credit or regulatory decision rule. Each annual financial-statement update could be converted into DPRI and assigned to low-, intermediate-, or high-resilience tertiles. Clinic administrators could use deterioration in DPRI or movement into the lowest tertile as a trigger for internal assessment of costs, debt, staffing, and liquidity planning. Public authorities could use aggregated low-tertile concentrations to identify segments that may require targeted support during shocks. Financial advisors and lenders could use the score as a screening input, while retaining human assessment and contextual information for final decisions.

The practical meaning of the reported metrics should therefore be understood in decision-support terms. An *R*^2^ near 0.64 in the crisis-informed recovery mode indicates that the model captures a substantial share of between-practice variation in 2021 DPRI, enough to prioritize follow-up but not enough to replace professional judgment. A balanced accuracy near 0.65 means that tertile classification is materially better than chance across low-, medium-, and high-resilience groups, but misclassification remains expected, especially near class boundaries. The finite-difference results also clarify scale: for example, increasing 2020 DPRI by 0.05 was associated with an average predicted 2021 DPRI increase of about 0.013, whereas isolated 1 percentage-point changes in profit margin produced much smaller changes. These values are predictive marginal effects, not causal elasticities.

At sector level, the framework may also support anticipatory monitoring. Practices with persistently weak DPRI values, unfavorable debt profiles, or deteriorating productivity may merit earlier managerial attention or selective support. The descriptive analysis reported in the Supplementary material points in the same direction: higher DPRI and higher turnover per employee were associated with lower failure rates and higher growth rates after the crisis. These findings are descriptive rather than causal, but they reinforce the practical value of resilience-oriented financial monitoring (22).

### 4.2 Study limitations

Several limitations should be acknowledged. First, the study is restricted to Bucharest dental practices, so institutional and market differences may limit generalizability to other countries or healthcare systems. Second, the predictors are purely accounting-based and do not capture service mix, ownership structure, patient demographics, clinical specialization, or management quality. Third, complete-case filtering improves internal consistency but may exclude firms with weaker reporting behavior or greater fragility. Fourth, tertile-based classification is useful for decision support, but it necessarily discards information relative to the continuous DPRI scale.

The added cohort-flow and imputation analyses make the complete-case limitation explicit. Complete-case firms were larger and more active than excluded firms, so the models should be interpreted as applying most directly to practices with stable financial reporting across the required years. Simple-imputation sensitivity preserved the main ordering of scenarios, but performance was lower in the enlarged imputed cohorts, which indicates that missingness and reporting instability are substantively relevant rather than merely technical.

Another limitation is that the models do not directly observe firm-level exposure to 2020–2021 policy support, deferrals, subsidies, or other crisis-management measures. Such interventions may have affected liquidity, staffing decisions, and reported profitability. Because these variables were not present in the extracted financial-statement dataset, the estimated associations should be interpreted as predictive relationships conditional on available accounting information, not as causal effects of financial structure alone.

The endpoint in 2021 is intentional. In the available repository extract, harmonized accounting fields are present for 2017–2021 only; 2020 is the shock year and 2021 is the first post-shock recovery year available for the current analysis. The study therefore evaluates crisis and immediate-recovery forecasting, not long-run post-pandemic convergence. A natural next step is an external temporal validation using harmonized 2022–2024 financial statements once they are integrated into the same data structure.

The synthetic recovery mode has an additional limitation: any error introduced in the first-stage 2020 forecast is carried into the second-stage 2021 prediction. In addition, the descriptive supplementary analysis defines failure as 2021 turnover below 30% of 2019 turnover. This threshold is an operational rule used for subgroup comparison and should not be interpreted as a regulatory or legal definition of failure.

## 5 Conclusion

This study developed and evaluated a financial-resilience forecasting framework for dental practices using the COVID-19 pandemic as a case study. The Dental Practice Resilience Index combined five fundamental financial dimensions into a single continuous outcome and allowed resilience to be examined across crisis-year, direct recovery, crisis-informed recovery, and synthetic recovery settings.

The results show that crisis-year forecasting from pre-crisis data is feasible, although moderate in difficulty, while direct recovery forecasting without 2020 information is more uncertain. Including observed 2020 data yielded the strongest and most stable results (*R*^2^ = 0.643 ± 0.071; balanced accuracy = 0.648 ± 0.044). The two-step synthetic recovery approach preserved part of the crisis-year signal, but it did not fully reproduce the benefit of observed crisis data.

Overall, the study highlights profitability, leverage control, multi-year stability, and crisis-period DPRI as the principal financial drivers of resilience. At the same time, it shows that resilience monitoring does not require opaque or excessively complex models: regularized linear models and tree-based ensembles both achieved competitive results. The proposed framework therefore offers a basis for systematic financial monitoring, early-warning assessment, and future extension to broader healthcare settings.

## Data Availability

The raw data supporting the conclusions of this article will be made available by the authors, without undue reservation.
