# Supplementary material for: Anticipating the economic resilience of dental practices in crises: a machine learning approach
Source: Front Public Health. 2026 Jun 30;14:1858605. doi: 10.3389/fpubh.2026.1858605 (PMC13367191; doi:10.3389/fpubh.2026.1858605)
Supplement: Supplementary file 1 [file Data_Sheet_1.pdf]

# Supplementary Material

## 1 APPENDIX A. COMPONENT TRANSFORMATION RULES AND DIAGNOSTICS

This appendix summarizes the transformation logic used to map the five DPRI components onto comparable 0–1 scales inside each evaluation split. The design emphasizes interpretability: the initial bounds are economically motivated, percentile clipping controls extreme values, and inversion is applied only when lower raw values correspond to stronger resilience.

**Table S1.** DPRI component transformation rules.

| Component | Initial bound | Training clip | Scaling | Inverted | Higher score means |
|---|---|---|---|---|---|
| Profit margin | $[-1, 1]$ | 5th–95th percentile | Min–max | No | Higher profitability |
| Expense efficiency | $[0, 2]$ | 5th–95th percentile | Min–max | Yes | Lower expense pressure |
| Debt-to-turnover | $[0, 5]$ | 5th–95th percentile | RobustScaler + min–max | Yes | Lower leverage |
| Turnover per employee | Positive support only | 2nd–98th percentile | RobustScaler + min–max | No | Higher labor productivity |
| Profit per employee | Non-zero support | 2nd–98th percentile | RobustScaler + min–max | No | Higher profit productivity |

**Table S2.** Mean transformation diagnostics across outer folds for the 2020 and 2021 cohorts.

| Cohort | Component | Low clip | High clip | Near 0 | Near 1 | SD |
|---|---|---|---|---|---|---|
| 2020 cohort | profit_margin | 0.05 | 0.05 | 0.0553 | 0.0732 | 0.2403 |
| 2020 cohort | expense_efficiency | 0.05 | 0.05 | 0.0553 | 0.0756 | 0.2364 |
| 2020 cohort | debt_to_turnover | 0.05 | 0.05 | 0.0541 | 0.4712 | 0.2624 |
| 2020 cohort | turnover_per_employee | 0.0206 | 0.0206 | 0.1447 | 0.0247 | 0.2402 |
| 2020 cohort | profit_per_employee | 0.0206 | 0.0206 | 0.0591 | 0.0241 | 0.1935 |
| 2021 cohort | profit_margin | 0.0499 | 0.0499 | 0.0584 | 0.0693 | 0.2507 |
| 2021 cohort | expense_efficiency | 0.0499 | 0.0499 | 0.0559 | 0.0687 | 0.2455 |
| 2021 cohort | debt_to_turnover | 0.0499 | 0.0499 | 0.0537 | 0.4881 | 0.2603 |
| 2021 cohort | turnover_per_employee | 0.0203 | 0.0203 | 0.1595 | 0.0243 | 0.2297 |
| 2021 cohort | profit_per_employee | 0.0203 | 0.0203 | 0.0983 | 0.0237 | 0.1981 |

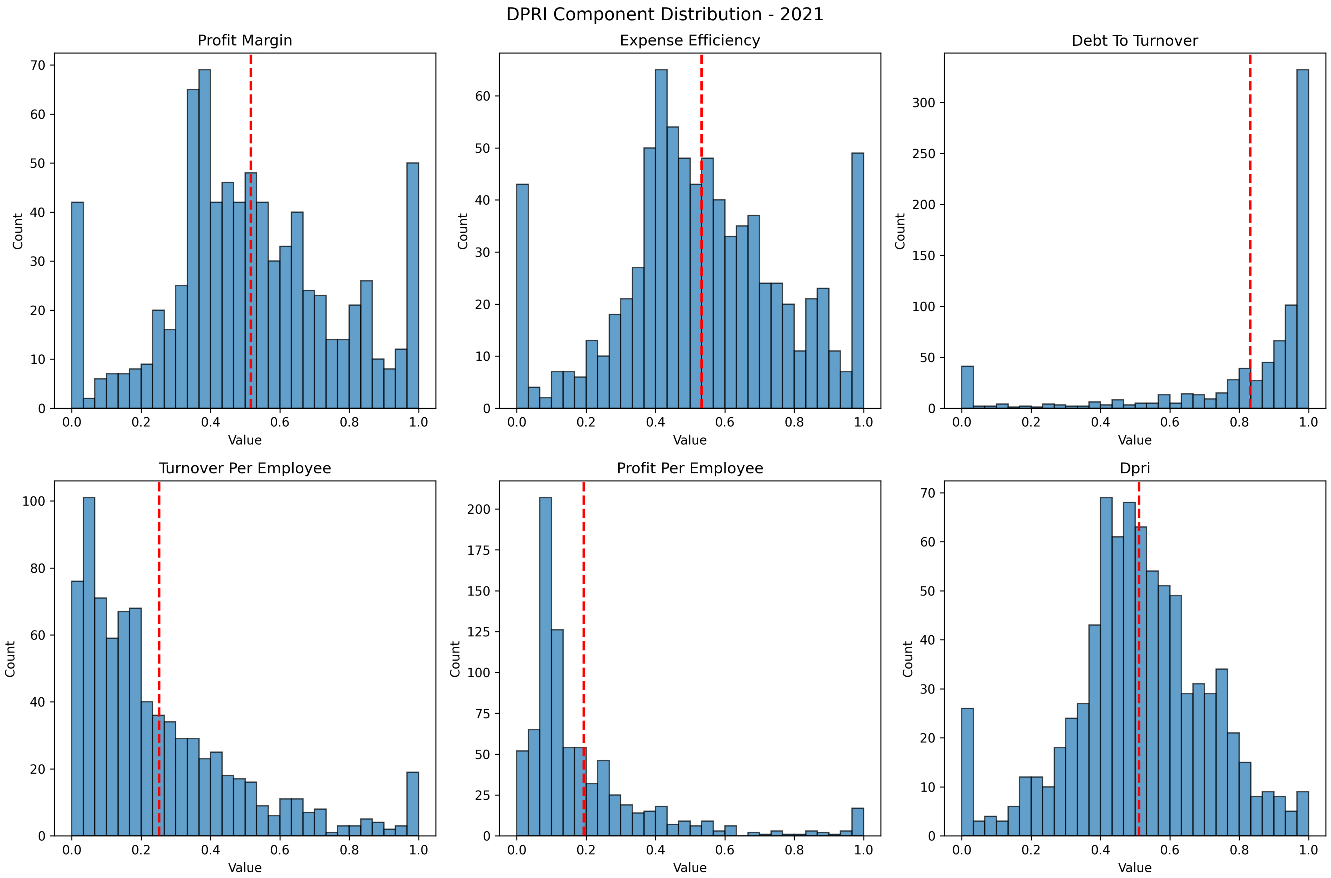


**Figure S1.** DPRI component distributions for the 2021 cohort after transformation.

## 2 APPENDIX A.2. ENGINEERED FEATURE FAMILIES AND BRANCH SELECTION

The predictive framework uses a broad engineered feature space composed of annual accounting ratios, scale terms, growth measures, temporal stability summaries, interaction terms, and composite engineered indicators. Table S3 summarizes the main feature families, while Table S4 shows the retained feature branch for each prediction mode.

**Table S3.** Main engineered feature families used in the predictive framework.

| Feature family | Examples | Purpose |
|---|---|---|
| Base annual ratios | dpri_2019, profit_margin_2019, debt_ratio_2019 | Level of profitability, leverage, and productivity |
| Scale and size | log_revenue_2017, log_debt_2018, log_employees_2017 | Practice scale and financial capacity |
| Growth terms | turnover_growth_2018_2019, employee_growth_2018_2019 | Short-run expansion or contraction |
| Stability terms | revenue_cv_3y, profit_stability_interaction, stability_score | Volatility and persistence across years |
| Interaction terms | size_efficiency_interaction, debt_profit_interaction | Non-additive relationships among financial dimensions |
| Composite engineered scores | financial_health, operational_excellence, resilience_composite | Aggregated summaries used alongside base ratios |

**Table S4.** Retained feature branch and model leaders by prediction mode.

| Mode | Cohort | Retained branch | Mean feature count | Best regression | Best classification |
|---|---|---|---|---|---|
| crisis-year forecasting (2020) | 850 | selected features | 22 | Linear Regression | Linear Regression |
| direct recovery forecasting (2021) without 2020 data | 801 | selected features | 30 | Random Forest | Linear Regression |
| recovery forecasting (2021) with observed 2020 data | 801 | all features | 99 | Elastic Net | Random Forest |
| synthetic recovery forecasting (2021) | 801 | all features | 79 | Elastic Net | Random Forest |

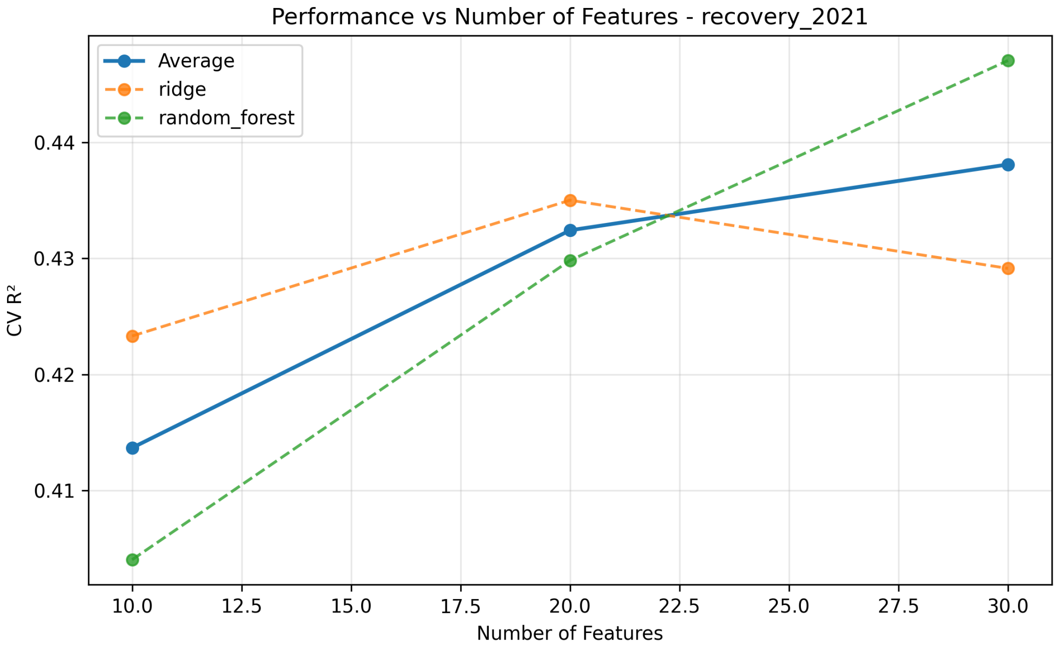


**Figure S2.** Cross-validated performance versus number of features for direct recovery forecasting without 2020 data.

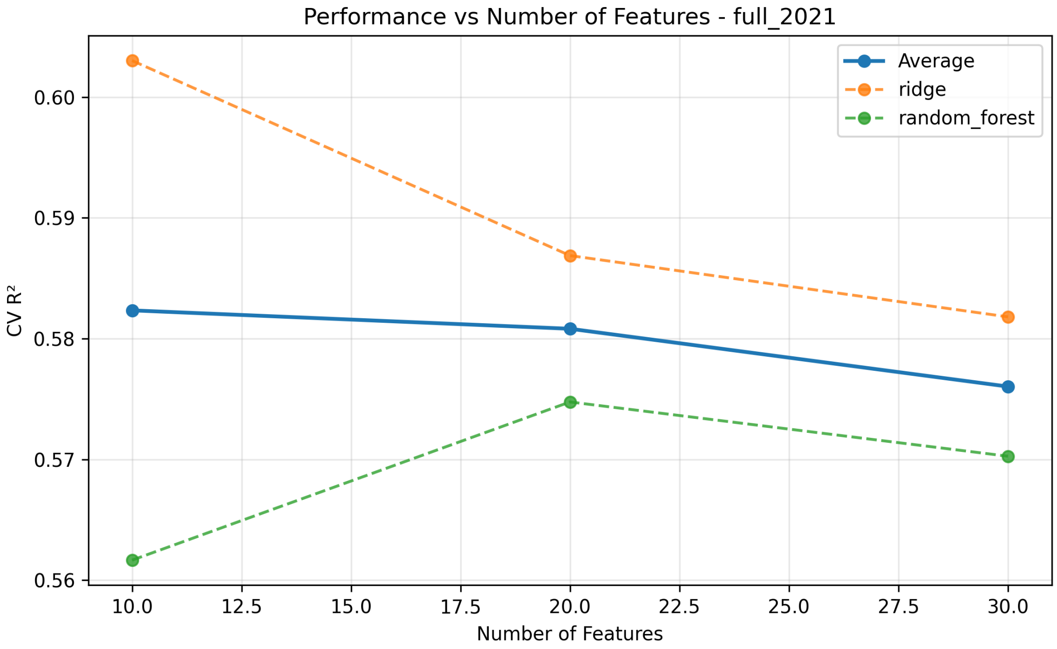


**Figure S3.** Cross-validated performance versus number of features for recovery forecasting with observed 2020 data.

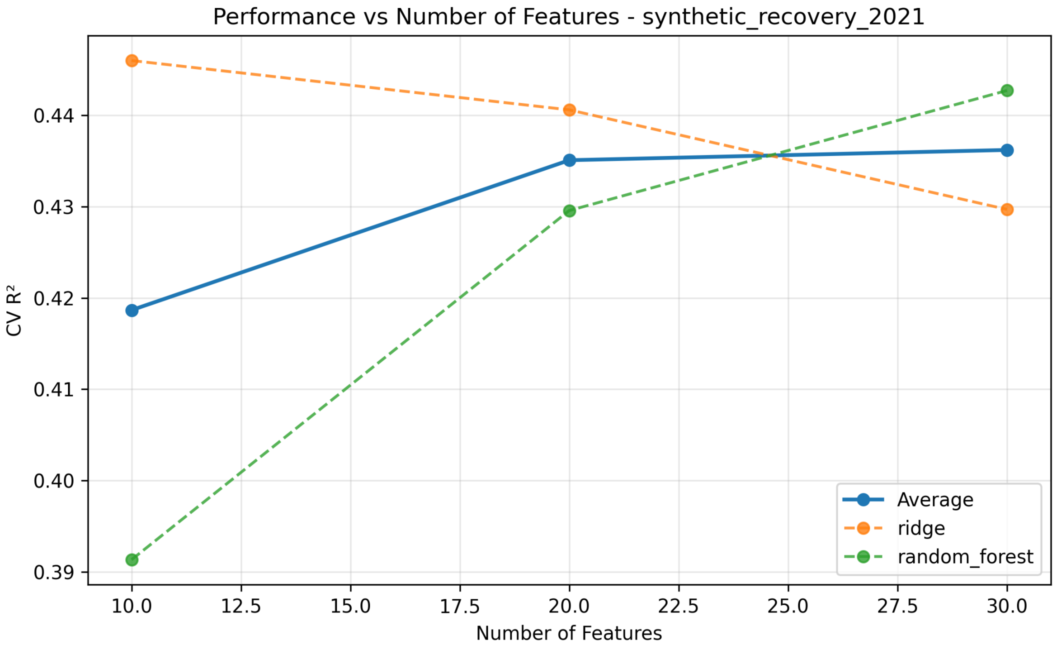


**Figure S4.** Cross-validated performance versus number of features for synthetic recovery forecasting.

# 3 APPENDIX A.3. TRAINING-TIME TERTILE BOUNDARY COMPUTATION

Derived classification was based on training-time tertile boundaries estimated from the DPRI distribution in each mode. Table S5 reports the low and high boundary values, their percentile positions, and the corresponding class prevalence in the training data.

**Table S5.** Training-time tertile boundaries and class prevalence by prediction mode.

| Mode | Method | Low boundary | High boundary | Low pct. | High pct. | T1 | T2 | T3 |
|---|---|---|---|---|---|---|---|---|
| crisis-year forecasting (2020) | GMM | 0.4528 | 0.5647 | 23.53 | 52.94 | 0.2353 | 0.2941 | 0.4706 |
| direct recovery forecasting (2021) without 2020 data | GMM | 0.4351 | 0.5411 | 37.34 | 60.94 | 0.3734 | 0.2359 | 0.3906 |
| recovery forecasting (2021) with observed 2020 data | GMM | 0.4351 | 0.5411 | 37.34 | 60.94 | 0.3734 | 0.2359 | 0.3906 |
| synthetic recovery forecasting (2021) | GMM | 0.4351 | 0.5411 | 37.34 | 60.94 | 0.3734 | 0.2359 | 0.3906 |

# 4 APPENDIX A.4. EXTENDED REGRESSION RESULTS ON THE FIXED 80/20 TEST SPLIT

The main text uses cross-validation as the basis for model comparison. The following tables report the full regression results obtained on the fixed 80/20 split used for visual interpretation.

**Table S6.** Regression results on the fixed 80/20 test split for crisis-year forecasting (2020).

| Model | $R^2$ | RMSE | MAE |
|---|---|---|---|
| Ridge | 0.5996 | 0.1185 | 0.0852 |
| Elastic Net | 0.5978 | 0.1187 | 0.0868 |
| Linear Regression | 0.5956 | 0.1191 | 0.0855 |
| Lasso | 0.5916 | 0.1197 | 0.088 |
| Random Forest | 0.5486 | 0.1258 | 0.091 |
| Gradient Boosting | 0.5247 | 0.1291 | 0.0939 |
| Extra Trees | 0.4965 | 0.1329 | 0.0944 |

**Table S7.** Regression results on the fixed 80/20 test split for direct recovery forecasting (2021) without 2020 data.

| Model | $R^2$ | RMSE | MAE |
|---|---|---|---|
| Random Forest | 0.527 | 0.1387 | 0.0991 |
| Elastic Net | 0.5143 | 0.1405 | 0.1045 |
| Lasso | 0.4986 | 0.1428 | 0.1062 |
| Ridge | 0.4974 | 0.143 | 0.1069 |
| Linear Regression | 0.4947 | 0.1434 | 0.1071 |
| Extra Trees | 0.4705 | 0.1467 | 0.1022 |
| Gradient Boosting | 0.4691 | 0.1469 | 0.1045 |

**Table S8.** Regression results on the fixed 80/20 test split for recovery forecasting (2021) with observed 2020 data.

| Model | $R^2$ | RMSE | MAE |
|---|---|---|---|
| Ridge | 0.7107 | 0.1085 | 0.0823 |
| Elastic Net | 0.7083 | 0.1089 | 0.0836 |
| Linear Regression | 0.7082 | 0.1089 | 0.0834 |
| Lasso | 0.7012 | 0.1102 | 0.0847 |
| Extra Trees | 0.6893 | 0.1124 | 0.0813 |
| Random Forest | 0.6872 | 0.1128 | 0.0808 |
| Gradient Boosting | 0.6794 | 0.1142 | 0.0819 |

**Table S9.** Regression results on the fixed 80/20 test split for synthetic recovery forecasting (2021).

| Model | $R^2$ | RMSE | MAE |
|---|---|---|---|
| Elastic Net | 0.5034 | 0.1421 | 0.1053 |
| Lasso | 0.4962 | 0.1431 | 0.1062 |
| Ridge | 0.4957 | 0.1432 | 0.1077 |
| Linear Regression | 0.4954 | 0.1433 | 0.1079 |
| Random Forest | 0.4895 | 0.1441 | 0.105 |
| Extra Trees | 0.4504 | 0.1495 | 0.107 |
| Gradient Boosting | 0.4371 | 0.1513 | 0.1107 |

# 5 APPENDIX A.5. EXTENDED CLASSIFICATION RESULTS AND DIAGNOSTIC SUMMARIES

The following tables report the full classification results for the fixed 80/20 split, followed by a compact residual and class-recall summary across the four modes.

**Table S10.** Classification results on the fixed 80/20 test split for crisis-year forecasting (2020).

| Model | Accuracy | Balanced Accuracy | F1 Score | Cohen Kappa |
|---|---|---|---|---|
| Linear Regression | 0.6412 | 0.6 | 0.6454 | 0.4325 |
| Ridge | 0.6353 | 0.5914 | 0.6398 | 0.4245 |
| Extra Trees | 0.6059 | 0.57 | 0.6164 | 0.3867 |
| Elastic Net | 0.6059 | 0.5596 | 0.6089 | 0.3746 |
| Lasso | 0.6 | 0.554 | 0.6038 | 0.3658 |
| Random Forest | 0.5882 | 0.5537 | 0.598 | 0.3565 |
| Gradient Boosting | 0.5882 | 0.5385 | 0.5958 | 0.3509 |

**Table S11.** Classification results on the fixed 80/20 test split for direct recovery forecasting (2021) without 2020 data.

| Model | Accuracy | Balanced Accuracy | F1 Score | Cohen Kappa |
|---|---|---|---|---|
| Extra Trees | 0.6646 | 0.6706 | 0.6707 | 0.4989 |
| Random Forest | 0.6335 | 0.6321 | 0.6395 | 0.4499 |
| Gradient Boosting | 0.6149 | 0.6264 | 0.6214 | 0.4302 |
| Ridge | 0.6149 | 0.6208 | 0.6234 | 0.4282 |
| Elastic Net | 0.6025 | 0.6161 | 0.6141 | 0.4164 |
| Linear Regression | 0.6087 | 0.6148 | 0.6181 | 0.4195 |
| Lasso | 0.5963 | 0.611 | 0.6089 | 0.4087 |

**Table S12.** Classification results on the fixed 80/20 test split for recovery forecasting (2021) with observed 2020 data.

| Model | Accuracy | Balanced Accuracy | F1 Score | Cohen Kappa |
|---|---|---|---|---|
| Gradient Boosting | 0.677 | 0.6673 | 0.684 | 0.5125 |
| Ridge | 0.6646 | 0.653 | 0.6753 | 0.4957 |
| Random Forest | 0.6584 | 0.6527 | 0.672 | 0.489 |
| Extra Trees | 0.646 | 0.6424 | 0.6609 | 0.4719 |
| Linear Regression | 0.646 | 0.6352 | 0.6593 | 0.4692 |
| Lasso | 0.6211 | 0.6234 | 0.6401 | 0.44 |
| Elastic Net | 0.6211 | 0.6179 | 0.639 | 0.4376 |

**Table S13.** Classification results on the fixed 80/20 test split for synthetic recovery forecasting (2021).

| Model | Accuracy | Balanced Accuracy | F1 Score | Cohen Kappa |
|---|---|---|---|---|
| Elastic Net | 0.6149 | 0.624 | 0.623 | 0.4299 |
| Extra Trees | 0.6087 | 0.6228 | 0.6135 | 0.4204 |
| Ridge | 0.6087 | 0.6125 | 0.6153 | 0.4174 |
| Lasso | 0.5963 | 0.611 | 0.6075 | 0.4081 |
| Gradient Boosting | 0.5963 | 0.611 | 0.5962 | 0.3995 |
| Random Forest | 0.5963 | 0.607 | 0.6052 | 0.4031 |
| Linear Regression | 0.6025 | 0.6041 | 0.6103 | 0.4079 |

**Table S14.** Residual summaries and tertile recalls for the best regression/classification test-split outputs.

| Mode | Test-split regression model | Residual mean | Residual SD | Recall T1 | Recall T2 | Recall T3 |
|---|---|---|---|---|---|---|
| crisis-year forecasting (2020) | Ridge | 0.0033 | 0.1184 | 49% | 54% | 77% |
| direct recovery forecasting (2021) without 2020 data | Random Forest | -0.0236 | 0.1367 | 62% | 70% | 70% |
| recovery forecasting (2021) with observed 2020 data | Ridge | -0.0106 | 0.1079 | 68% | 57% | 75% |
| synthetic recovery forecasting (2021) | Elastic Net | -0.021 | 0.1406 | 51% | 65% | 71% |

# 6 APPENDIX A.6. NUMERIC FEATURE RANKINGS

The main text now presents SHAP-based feature-attribution profiles as the primary explainability figure. The following tables retain the numeric top-10 model-specific feature rankings from the fixed 80/20 diagnostic split, using native tree importances where available and absolute standardized coefficients for linear or regularized models.

**Table S15.** Top 10 features for crisis-year forecasting (2020).

| Feature | Importance |
|---|---|
| stability_score | 0.1156 |
| financial_health | 0.0814 |
| debt_to_turnover_2019 | 0.0589 |
| debt_ratio_2019 | 0.0589 |
| profit_stability_interaction | 0.0483 |
| size_efficiency_interaction | 0.0408 |
| growth_stability_interaction | 0.0392 |
| dpri_2018 | 0.0384 |
| leverage_squared | 0.037 |
| debt_profit_interaction | 0.0358 |

**Table S16.** Top 10 features for direct recovery forecasting (2021) without 2020 data.

| Feature | Importance |
|---|---|
| dpri_2019 | 0.1334 |
| size_efficiency_interaction | 0.1025 |
| operational_excellence | 0.082 |
| profit_per_employee_2019 | 0.0513 |
| financial_health | 0.0457 |
| debt_to_turnover_2019 | 0.0375 |
| resilience_composite | 0.0351 |
| debt_ratio_2019 | 0.0343 |
| leverage_risk | 0.0325 |
| expense_ratio_2019 | 0.029 |

**Table S17.** Top 10 features for recovery forecasting (2021) with observed 2020 data.

| Feature | Importance |
|---|---|
| dpri_2020 | 0.1036 |
| dpri_2018 | 0.0624 |
| profit_margin_2017 | 0.0578 |
| size_efficiency_interaction | 0.0565 |
| log_revenue_2017 | 0.0563 |
| profit_margin_2020 | 0.0554 |
| dpri_2017 | 0.0542 |
| log_employees_2017 | 0.0425 |
| growth_stability_interaction | 0.0413 |
| profit_per_employee_2020 | 0.0377 |

**Table S18.** Top 10 features for synthetic recovery forecasting (2021).

| Feature | Importance |
|---|---|
| predicted_dpri_2020 | 0.0979 |
| profit_per_employee_2019 | 0.0214 |
| revenue_cv_3y | 0.0095 |
| risk_adjusted_return | 0.0082 |
| efficiency_squared | 0.005 |
| debt_to_turnover_2018 | 0.0049 |
| log_debt_2018 | 0.0036 |
| debt_ratio_2018 | 0.0036 |
| log_debt_2017 | 0.0034 |
| profit_improved_2017_2018 | 0.003 |

# 7 APPENDIX A.7. SECONDARY DESCRIPTIVE ANALYSIS BY EMPLOYEE-SIZE SUBGROUP

For descriptive comparison, DPRI was evaluated alongside turnover per employee in the subgroup of firms with complete 2019–2021 data. Firms were split by mean employee count ($\leq 3$ versus $> 3$ employees), failure was defined as 2021 turnover below 30% of 2019 turnover, and growth was defined as 2021 turnover above 2019 turnover. Turnover per employee was retained as a benchmark because it has previously been examined as a dental-practice indicator in its own right.

In this descriptive cohort, smaller practices had a failure rate of 1.54% and a growth rate of 69.47%, whereas larger practices had a failure rate of 0.35% and a growth rate of 76.33%. Mean 2020 DPRI was 0.533 in the smaller-practice group and 0.542 in the larger-practice group.

**Table S19.** Descriptive subgroup summary by employee-size group.

| Employee-size group | n | Failure rate 2021 | Growth rate 2021 | Mean 2020 DPRI | Mean 2020 turnover per employee |
|---|---|---|---|---|---|
| $\leq 3$ employees | 714 | 1.54% | 69.47% | 0.533 | 162,008.91 |
| $> 3$ employees | 283 | 0.35% | 76.33% | 0.542 | 196,798.60 |

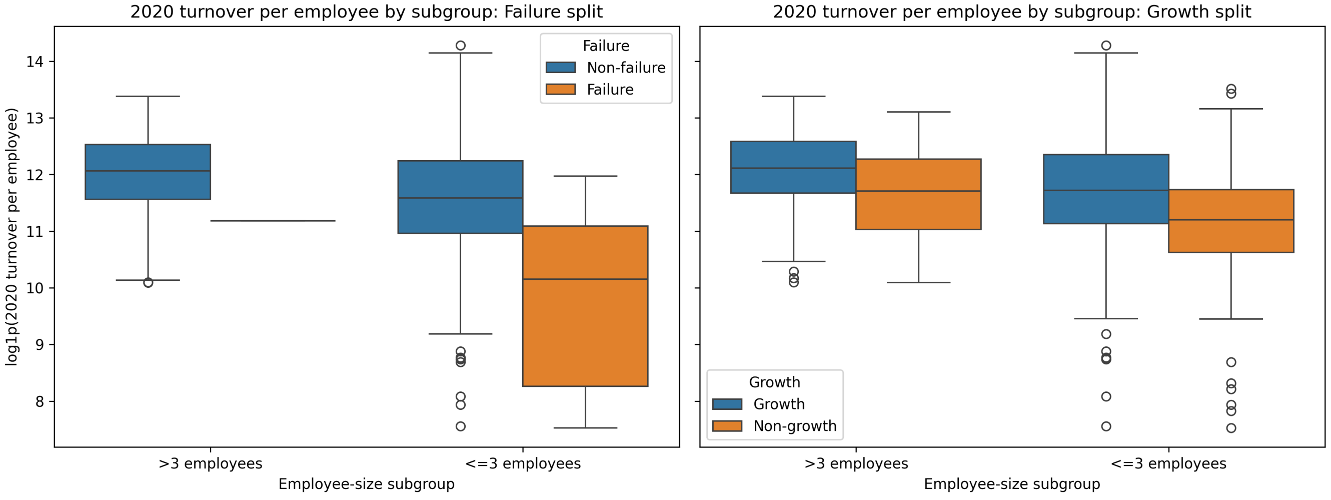


**Figure S5.** Failure and growth patterns by turnover per employee, stratified by employee-size subgroup.

These subgroup figures should be interpreted as descriptive complements to the main predictive analysis. They provide intuition about post-crisis patterns by practice size, but they do not replace the cross-validated results reported in the main manuscript.

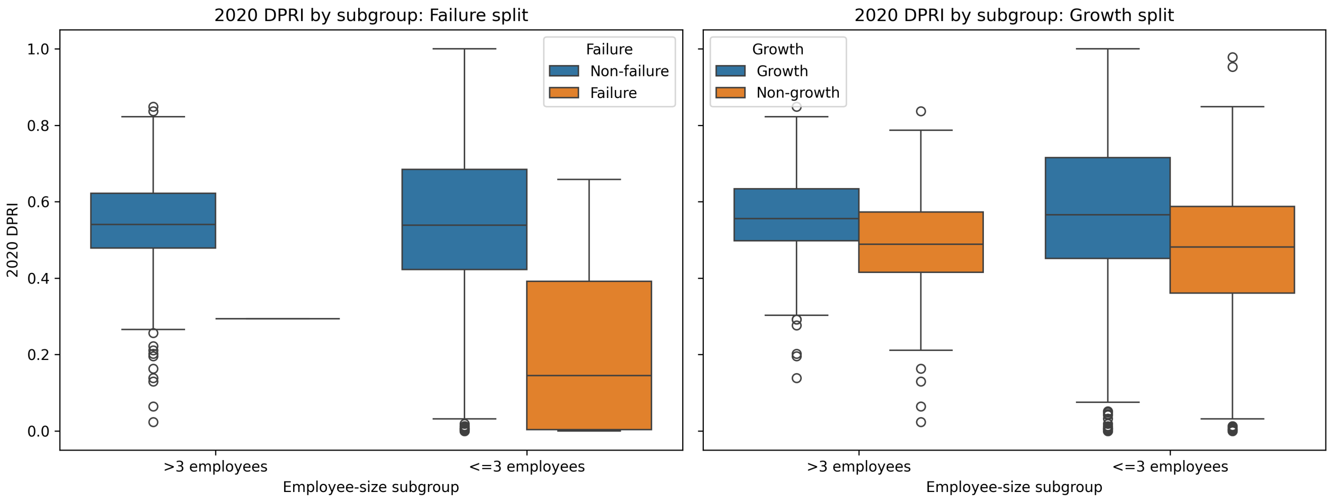


**Figure S6.** Failure and growth patterns by DPRI, stratified by employee-size subgroup.

# 8 APPENDIX A.8. ADDITIONAL SENSITIVITY AND ROBUSTNESS ANALYSES

This appendix section records additional analyses used to assess methodological transparency, sample representativeness, complete-case filtering, DPRI construction, model-ranking uncertainty, explainability, traditional accounting-ratio benchmarks, and robustness across random seeds and subsamples. These outputs complement the primary cross-validation tables in the main manuscript.

Table S20 is an index of the methodological audit. It is included first so that each subsequent table and figure can be traced back to the methodological purpose it addresses.

**Table S20.** Methodological audit-output map showing each additional analysis family, the main output, and the methodological purpose addressed.

| Audit family | Main output | Methodological purpose addressed |
|---|---|---|
| Cohort representativeness | Cohort flow, exclusion summaries, and standardized mean differences | Sample drop from 2474 firms to the complete-case cohorts. |
| Complete-case sensitivity | Complete-case versus simple-imputation model metrics | Whether results depend on complete-case filtering. |
| DPRI transparency | Component dictionary, component distributions, and equal-weight sensitivity | Transparency and robustness of the composite-index construction. |
| Model comparison | Paired metric differences and confidence intervals | Whether small model differences justify strong superiority claims. |
| Explainability and finite differences | Permutation importance and predictive marginal effects | Which financial drivers matter and how large their predictive effects are. |
| Robustness | Repeated random seeds and subsample summaries | Stability of conclusions under resampling. |

Table S21 documents how the analytic cohorts were formed from the initial extract. The target-computable column distinguishes firms for which the outcome DPRI could be calculated from firms that also met all complete-case predictor-history requirements.

Table S22 summarizes the representativeness check for the latest pre-crisis year common to all prediction modes. The standardized mean differences show that complete-case firms were generally larger and more financially active than excluded firms, which is why the limitation is explicitly discussed in the main manuscript.

**Table S21.** Cohort-flow audit from the initial Ministry of Finance extract to the target-computable and complete-case cohorts used in the four prediction modes.

| Prediction mode | Initial firms | Target year | Feature years | Target computable | Complete case | Complete-case share |
|---|---|---|---|---|---|---|
| Crisis-year forecasting | 2474 | 2020 | 2017–2019 | 1233 | 850 | 34.36% |
| Direct recovery forecasting | 2474 | 2021 | 2017–2019 | 1287 | 801 | 32.38% |
| Crisis-informed recovery forecasting | 2474 | 2021 | 2017–2020 | 1287 | 801 | 32.38% |
| Synthetic crisis-bridge recovery forecasting | 2474 | 2021 | 2017–2019 | 1287 | 801 | 32.38% |

**Table S22.** Included-versus-excluded representativeness summary for 2019, the latest pre-crisis year used in all prediction modes, with standardized mean differences for key accounting variables.

| Prediction cohort | 2019 variable | Included mean | Excluded mean | Standardized mean difference |
|---|---|---|---|---|
| Crisis-year forecasting | Turnover | 809,296 | 154,510 | 0.334 |
| Crisis-year forecasting | Profit | 148,751 | 50,420 | 0.368 |
| Crisis-year forecasting | Employees | 4.08 | 0.66 | 0.548 |
| 2021 recovery cohorts | Turnover | 826,627 | 173,998 | 0.324 |
| 2021 recovery cohorts | Profit | 152,442 | 52,413 | 0.367 |
| 2021 recovery cohorts | Employees | 4.15 | 0.78 | 0.526 |

Table S23 reports the key cleaning counts behind the eligibility rules. Negative profit is retained as an economically meaningful loss signal, whereas zero or missing turnover and employee counts invalidate ratio denominators and therefore drive complete-case exclusions.

**Table S23.** Database-cleaning audit summary showing retained negative-profit observations and denominator problems that affect turnover-based and employee-based ratios.

| Year | Negative profit retained | Zero turnover | Missing turnover | Zero employees | Missing employees |
|---|---|---|---|---|---|
| 2017 | 400 | 124 | 1055 | 358 | 1055 |
| 2018 | 451 | 151 | 884 | 462 | 884 |
| 2019 | 454 | 150 | 739 | 567 | 739 |
| 2020 | 485 | 185 | 627 | 587 | 627 |
| 2021 | 411 | 194 | 485 | 671 | 485 |

Table S24 evaluates whether the DPRI conclusions depend on the primary weighting scheme. The high correlations between the primary DPRI and alternative definitions indicate that the empirical ranking of practices is not fragile to equal weighting or leave-one-component-out variants.

**Table S24.** DPRI weighting-sensitivity analysis comparing the primary weighted index with equal-weight and leave-one-component-out alternatives.

| Year | Alternative definition | Pearson correlation | Spearman correlation |
|---|---|---|---|
| 2020 | Equal weights | 0.990 | 0.994 |
| 2020 | Leave out profit margin | 0.984 | 0.984 |
| 2020 | Leave out expense efficiency | 0.991 | 0.988 |
| 2020 | Leave out debt-to-turnover | 0.976 | 0.981 |
| 2020 | Leave out turnover per employee | 0.988 | 0.980 |
| 2020 | Leave out profit per employee | 0.998 | 0.999 |
| 2021 | Equal weights | 0.989 | 0.993 |
| 2021 | Leave out profit margin | 0.980 | 0.981 |
| 2021 | Leave out expense efficiency | 0.990 | 0.989 |
| 2021 | Leave out debt-to-turnover | 0.976 | 0.983 |
| 2021 | Leave out turnover per employee | 0.989 | 0.987 |
| 2021 | Leave out profit per employee | 0.998 | 0.999 |

Table S25 reports the complete-case versus simple-imputation sensitivity analysis. The imputed-predictor cohorts relax complete predictor-history requirements while retaining the requirement that target-year DPRI be computable, allowing the robustness of the main scenario ordering to be assessed in a broader sample.

**Table S25.** Complete-case versus simple-imputation sensitivity analysis comparing model performance and interpretation across the four prediction modes.

| Prediction mode | Complete-case best result | Imputed-predictor best result | Interpretation |
|---|---|---|---|
| Crisis-year forecasting | $n = 850$; Elastic Net $R^2 = 0.544$, balanced accuracy $= 0.659$ | $n = 1233$; Elastic Net $R^2 = 0.482$, balanced accuracy $= 0.640$ | Predictive signal remains, with attenuation after inclusion of less complete firms. |
| Direct recovery forecasting | $n = 801$; Random Forest $R^2 = 0.443$, balanced accuracy $= 0.612$ | $n = 1287$; Random Forest balanced accuracy $= 0.564$; Elastic Net $R^2 = 0.350$ | Recovery without 2020 remains the weakest scenario. |
| Crisis-informed recovery forecasting | $n = 801$; Elastic Net $R^2 = 0.631$, balanced accuracy $= 0.674$ | $n = 1287$; Elastic Net $R^2 = 0.561$, balanced accuracy $= 0.636$ | Observed 2020 information remains the strongest configuration. |
| Synthetic crisis-bridge recovery forecasting | $n = 801$; Ridge $R^2 = 0.470$, balanced accuracy $= 0.618$ | $n = 1287$; Random Forest balanced accuracy $= 0.576$; Elastic Net $R^2 = 0.350$ | The bridge recovers partial information but does not match observed 2020 data. |

Table S26 condenses the model-comparison, explainability, and finite-difference checks. It is intended to support cautious model-ranking language and to show which financial signals drive predictions, not to replace the primary cross-validation tables in the main manuscript.

**Table S26.** Additional model-comparison, repeated-seed, permutation-importance, and finite-difference checks used to interpret model rankings and financial drivers.

| Prediction mode | Top-model separation in added paired comparison | Repeated-seed pattern | Leading permutation-importance signal | Finite-difference interpretation |
|---|---|---|---|---|
| Crisis-year forecasting | Lasso exceeded Elastic Net by only $\Delta R^2 = 0.003$ | Elastic Net ranked first in 5/5 seed runs | 2019 DPRI, followed by size-efficiency interaction | A 1 percentage-point increase in 2019 profit margin changed predicted DPRI by about $+0.0003$. |
| Direct recovery forecasting | Extra Trees exceeded Lasso by only $\Delta R^2 < 0.001$ | Random Forest ranked first in 3/5 seed runs, Ridge in 2/5 | 2019 DPRI and size-efficiency interaction | Increasing 2019 DPRI by 0.05 changed predicted 2021 DPRI by about $+0.0057$. |
| Crisis-informed recovery forecasting | Lasso exceeded Elastic Net by only $\Delta R^2 = 0.009$ | Elastic Net ranked first in 4/5 seed runs | 2020 DPRI was dominant | Increasing 2020 DPRI by 0.05 changed predicted 2021 DPRI by about $+0.0134$. |
| Synthetic crisis-bridge recovery forecasting | Ridge exceeded Elastic Net by only $\Delta R^2 = 0.008$ | Ridge ranked first in 4/5 seed runs | 2019 DPRI, size-efficiency interaction, and predicted 2020 DPRI | A 1 percentage-point increase in 2019 profit margin changed predicted 2021 DPRI by about $+0.0011$. |

Table S27 compares models based only on traditional accounting ratios with models using the full engineered feature set. This comparison shows how much of the predictive signal is already captured by conventional financial ratios and helps position the machine-learning framework relative to standard accounting-ratio approaches.

**Table S27.** Traditional accounting-ratio baselines compared with full engineered feature models for the main model family used in each prediction setting.

| Prediction mode | Model family | Ratio-baseline $R^2$ | Full engineered $R^2$ |
|---|---|---|---|
| Crisis-year forecasting | Ridge | 0.535 | 0.527 |
| Direct recovery forecasting | Random Forest | 0.430 | 0.443 |
| Crisis-informed recovery forecasting | Random Forest | 0.619 | 0.629 |
| Synthetic crisis-bridge recovery forecasting | Ridge | 0.421 | 0.470 |

Table S28 summarizes whether model rankings were stable when the analysis was repeated with different random seeds. The first-rank count records how often each model ranked first, while the final column records how often it remained practically close to the best model.

**Table S28.** Repeated-seed rank-stability summary showing mean rank, first-rank frequency, and practical closeness to the best model.

| Prediction mode | Model | Mean rank | First-rank count | Within 0.01 of best count |
|---|---|---|---|---|
| Crisis-year forecasting | Elastic Net | 1.0 | 5 | 5 |
| Crisis-year forecasting | Ridge | 2.2 | 0 | 1 |
| Crisis-year forecasting | Random Forest | 2.8 | 0 | 1 |
| Direct recovery forecasting | Random Forest | 1.8 | 3 | 3 |
| Direct recovery forecasting | Elastic Net | 2.0 | 0 | 5 |
| Direct recovery forecasting | Ridge | 2.2 | 2 | 3 |
| Crisis-informed recovery forecasting | Elastic Net | 1.2 | 4 | 4 |
| Crisis-informed recovery forecasting | Random Forest | 2.4 | 1 | 2 |
| Crisis-informed recovery forecasting | Ridge | 2.4 | 0 | 2 |
| Synthetic crisis-bridge recovery forecasting | Ridge | 1.4 | 4 | 4 |
| Synthetic crisis-bridge recovery forecasting | Elastic Net | 1.8 | 1 | 4 |
| Synthetic crisis-bridge recovery forecasting | Random Forest | 2.8 | 0 | 0 |

Table S29 clarifies the empirical endpoint. The harmonized repository extract used for this analysis contains fields for 2017–2021, so the study is framed as a crisis-year and first-recovery-year analysis rather than a long-run 2022–2024 follow-up.

**Table S29.** Available-year audit documenting the calendar years and accounting fields present in the harmonized repository extract used for the analysis.

| Years present in harmonized extract | Accounting fields present each year |
|---|---|
| 2017–2021 | Turnover, profit, employees, debt, revenue, expenses |

Figures S7–S12 provide graphical versions of the main sensitivity and robustness checks. They are placed after the tables so that readers can first inspect the exact tabular summaries and then use the figures for visual interpretation.

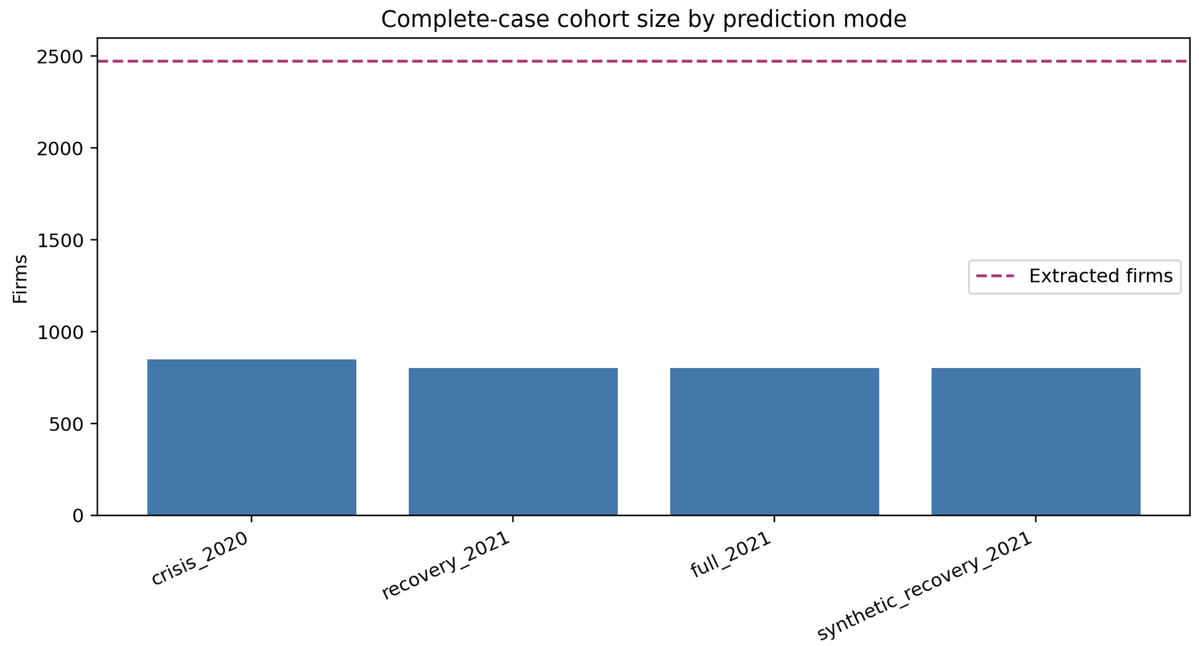


**Figure S7.** Cohort-flow audit from the initial extract to target-computable and complete-case analytic cohorts.

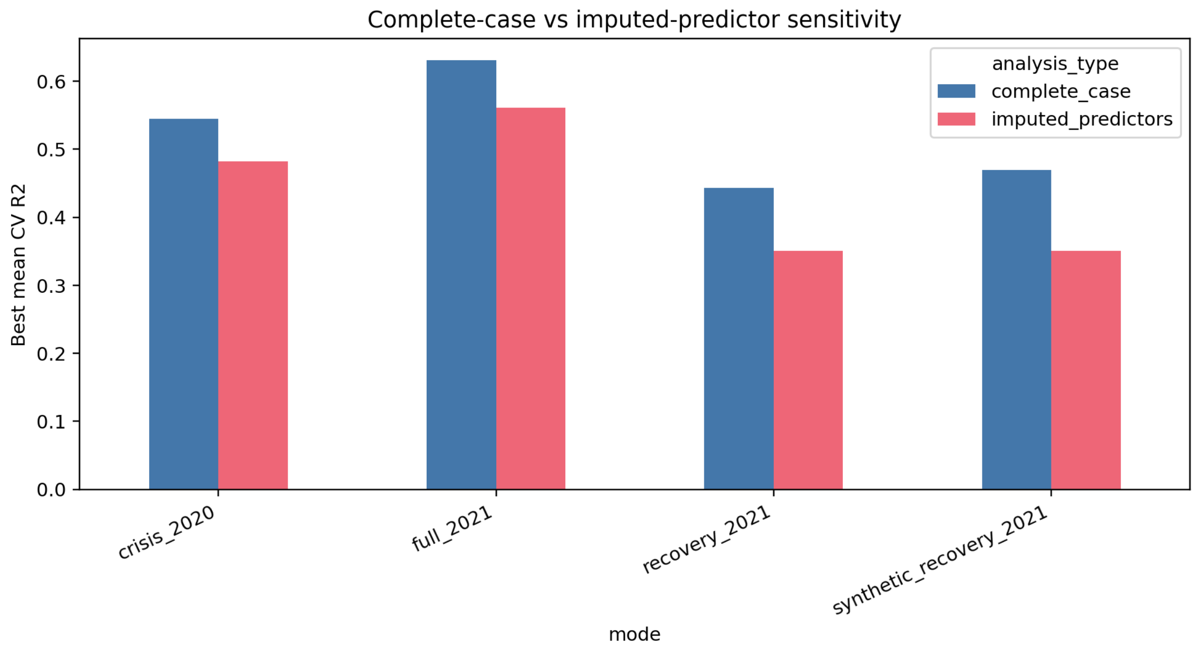


**Figure S8.** Complete-case versus simple-imputation sensitivity results across prediction modes.

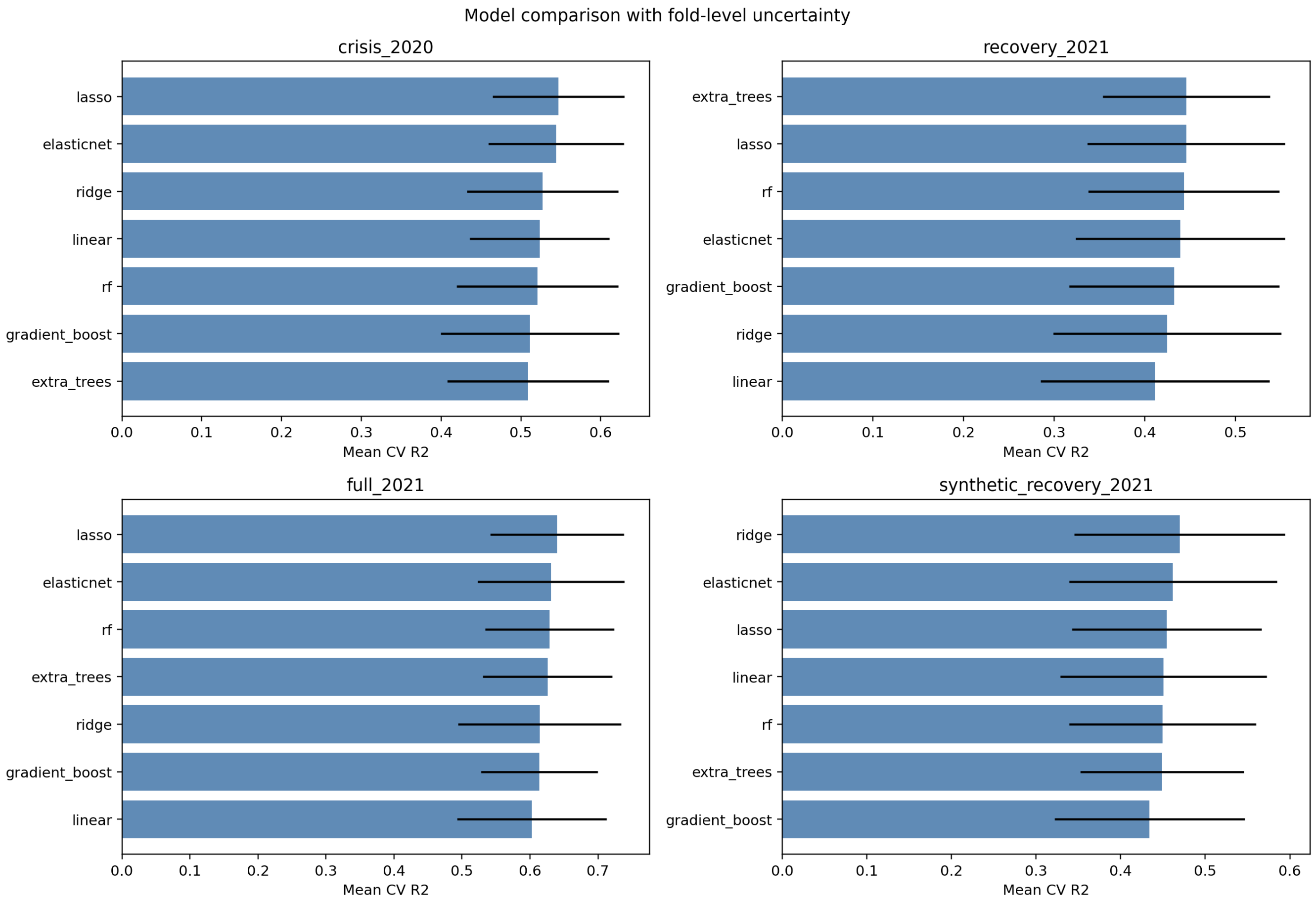


**Figure S9.** Paired model-comparison intervals used to support cautious model-ranking language.

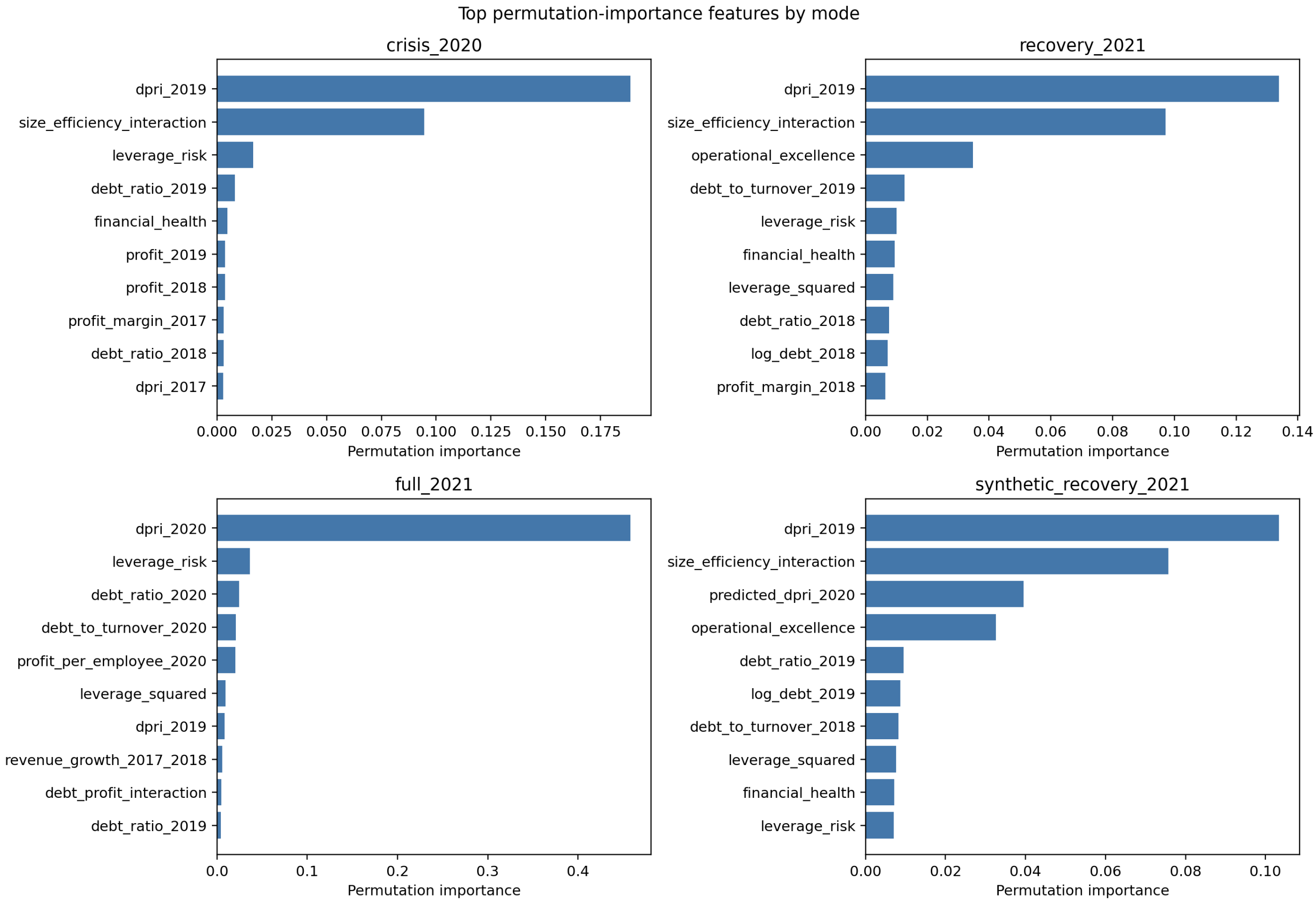


**Figure S10.** Model-agnostic permutation-importance summary for explainability.

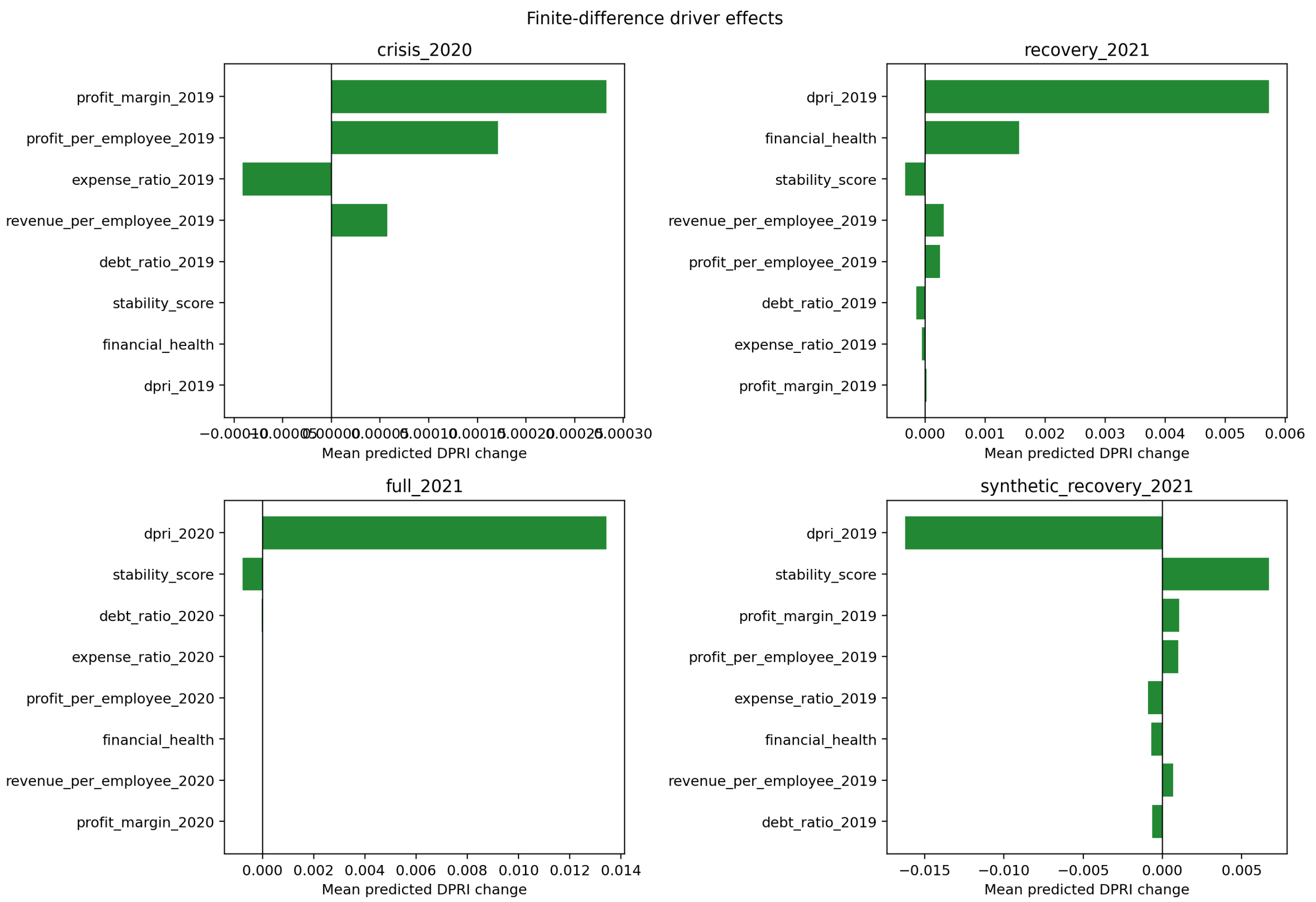


**Figure S11.** Finite-difference predictive effects used to quantify the scale of financial drivers.

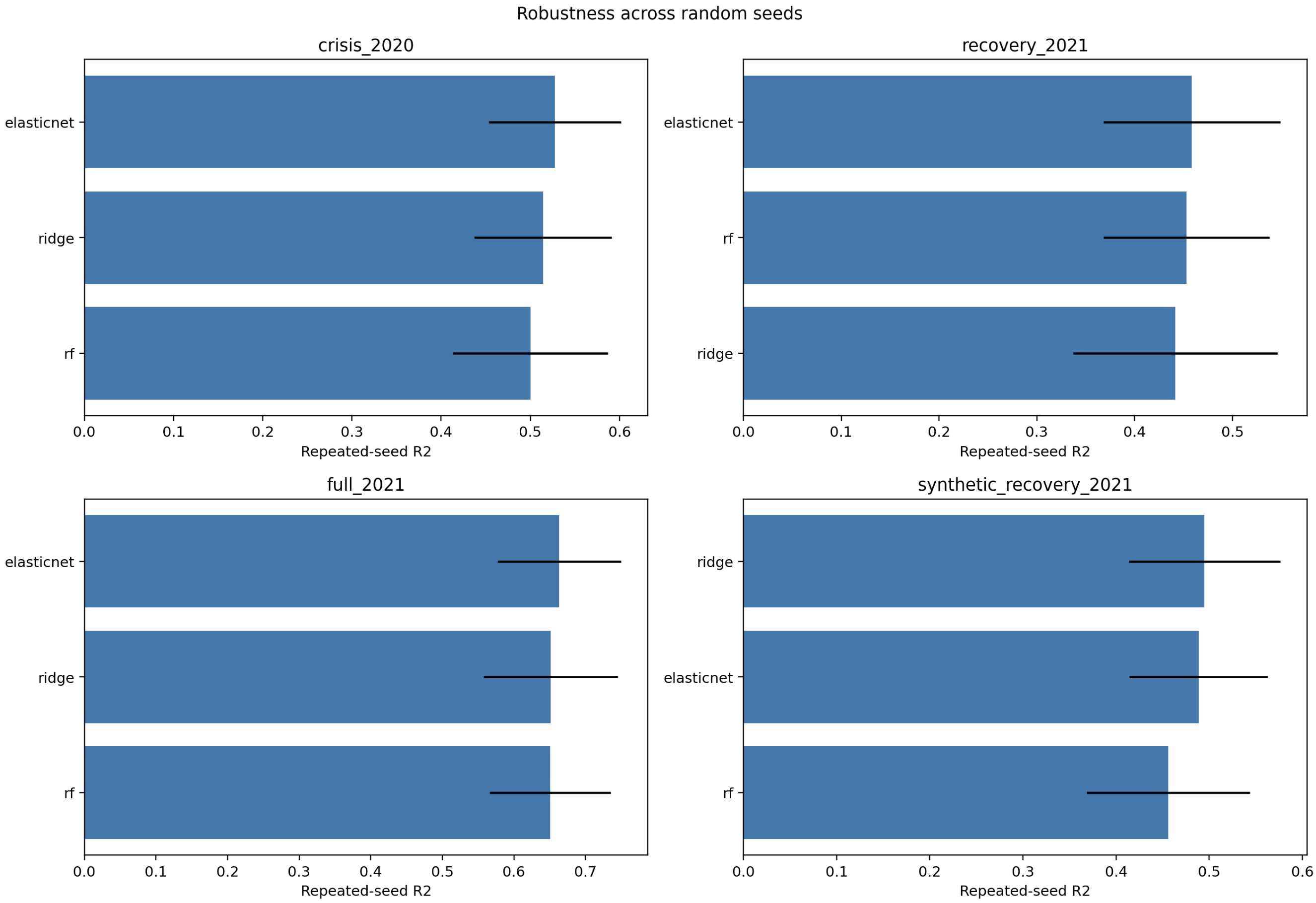


**Figure S12.** Repeated-seed and subsample robustness summary for the main prediction modes.
